# In Vitro and In Vivo Drug-Response Profiling Using Patient-Derived High-Grade Glioma

**DOI:** 10.3390/cancers15133289

**Published:** 2023-06-22

**Authors:** Robin G. Rajan, Virneliz Fernandez-Vega, Jantzen Sperry, Jonathan Nakashima, Long H. Do, Warren Andrews, Simina Boca, Rezwanul Islam, Sajeel A. Chowdhary, Jan Seldin, Glauco R. Souza, Louis Scampavia, Khalid A. Hanafy, Frank D. Vrionis, Timothy P. Spicer

**Affiliations:** 1 Helene and Stephen Weicholz Cell Therapy Laboratory, Marcus Neuroscience Institute, Boca Raton Regional Hospital, 800 Meadows Road, Boca Raton, FL 33486, USA; robin.rajan@baptisthealth.net (R.G.R.); schowdhary.md@gmail.com (S.A.C.); khanafy@health.fau.edu (K.A.H.); 2 The Herbert Wertheim UF Scripps Institute Molecular Screening Center, Department of Molecular Medicine, UF Scripps Biomedical Research, 130 Scripps Way, Jupiter, FL 33458, USA; vfernandezvega@ufl.edu (V.F.-V.); scampl@ufl.edu (L.S.); 3 Certis Oncology, 5626 Oberlin Dr. Suite 110, San Diego, CA 92121, USA; jsperry@certisoncology.com (J.S.); nakashima@certisoncology.com (J.N.); ldo@certisoncology.com (L.H.D.); wandrews@certisoncology.com (W.A.); 4 Innovation Center for Biomedical Informatics (ICBI), Departments of Oncology and Biostatistics, Bioinformatics and Biomathematics, Georgetown University Medical Center, 2115 Wisconsin Ave NW, Suite G100, Washington, DC 20007, USA; simina.m.boca@gmail.com; 5 Florida Atlantic University College of Medicine, 777 Glades Road, Boca Raton, FL 33431, USA; islamr@health.fau.edu; 6 Greiner Bio-One North America, Inc., 4238 Capital Drive, Monroe, NC 28110, USA; jan.seldin@gbo.com (J.S.); glauco.souza@gbo.com (G.R.S.)

**Keywords:** organoid, GBM, cancer, phenotypic, high-throughput screening (HTS)

## Abstract

**Simple Summary:**

To date, personalized and comprehensive approaches to combat treatment resistances and failures are limited due to the highly heterogeneous, resistive, and invasive phenotype of GBM tumors. We present an integrative genomic, in vitro, and in vivo functional treatment paradigm for GBM. Our study utilizes patient-derived 3D organoids, as they have better concordance with the parent tumor for the in vitro assays and in vivo PDX mouse model. In vitro HTS of the 3D organoids for effective drugs combined with RNAseq analysis to identify differentially enriched genomic pathways and gene targets has enabled rapid generation of clinically relevant information. When supplemented with validation using in vivo PDX mouse models of tumor growth, this creates a robust precision medicine paradigm. Rapidly implemented individualized drug response prediction models thus provide actionable information for the physician to combat recurrences or treatment resistances in GBM. Moreover, it is a scalable workflow, which includes compounds not yet approved for GBM but showing promise in clinical trials.

**Abstract:**

Background: Genomic profiling cannot solely predict the complexity of how tumor cells behave in their in vivo microenvironment and their susceptibility to therapies. The aim of the study was to establish a functional drug prediction model utilizing patient-derived GBM tumor samples for in vitro testing of drug efficacy followed by in vivo validation to overcome the disadvantages of a strict pharmacogenomics approach. Methods: High-throughput in vitro pharmacologic testing of patient-derived GBM tumors cultured as 3D organoids offered a cost-effective, clinically and phenotypically relevant model, inclusive of tumor plasticity and stroma. RNAseq analysis supplemented this 128-compound screening to predict more efficacious and patient-specific drug combinations with additional tumor stemness evaluated using flow cytometry. In vivo PDX mouse models rapidly validated (50 days) and determined mutational influence alongside of drug efficacy. We present a representative GBM case of three tumors resected at initial presentation, at first recurrence without any treatment, and at a second recurrence following radiation and chemotherapy, all from the same patient. Results: Molecular and in vitro screening helped identify effective drug targets against several pathways as well as synergistic drug combinations of cobimetinib and vemurafenib for this patient, supported in part by in vivo tumor growth assessment. Each tumor iteration showed significantly varying stemness and drug resistance. Conclusions: Our integrative model utilizing molecular, in vitro, and in vivo approaches provides direct evidence of a patient’s tumor response drifting with treatment and time, as demonstrated by dynamic changes in their tumor profile, which may affect how one would address that drift pharmacologically.

## 1. Introduction

Glioblastoma multiforme (GBM) is the most common and aggressive primary brain cancer with the median survival in the United States reported as 15 months, and a 5-year survival rate estimated at a dismal 5% for adults 45 years and older [1,2]. A recent systematic review indicates no current systemic treatment for recurrent GBM that affords overall survival (OS) benefits [3]. Treatment resistance after surgical resection develops in spite of multimodal approaches, including chemotherapy with temozolomide (TMZ) and high-dose radiation, possibly due to high intra- and inter-tumor heterogeneity with a variety of genetic and epigenetic mutations associated with GBM [4,5].

Traditional 2D cell culture systems fail to accurately mimic the genetic and environmental complexity of GBM with regard to its extracellular matrix (ECM), clonal survival, and mutational profile representing the parent tumor [6]. The 3D organoid models from GBM cell lines and patient-derived organoids, when used to successfully test effective pharmacological compounds, have demonstrated considerable variability in drug response [7,8]. It is now widely recognized that 3D organoid models more accurately recapitulate inter-and intra-tumoral heterogeneity while maintaining many key features of GBM, such as its cytoarchitecture and cell-cell interactions [9]. Patient-derived glioblastoma stem cell (GSC) cultures and GBM organoids (GBO) are better mimics of cellular and mutational diversity observed in parental tumors, yielding improved drug-screening strategies [10,11]. Furthermore, obtaining in vitro models as an accurate representation of the parental tumor is necessary in order to achieve and correctly assess effective personalized treatment strategies, such as patient-specific immunotherapies like CAR T-cells [12,13].

Cell line-based murine models have failed to recapitulate the molecular, histological, and cellular characteristics of the primary human tumor, resulting in poor translation to clinical development. A more accurate pre-clinical mouse model such as PDX or the genetically engineered mouse model (GEMM) is essential to validate in vitro drug sensitivities and more likely to ensure clinically relevant therapeutic information [14,15,16,17]. In addition to the mutational burden during tumor development, GBMs, like hematopoietic malignancies and other solid tumors, have been shown to contain a small population of cancer stem cells known as glioma stem cells (GSCs), with the capacity to reconstitute the heterogeneity of the parental tumor [18,19,20]. Moreover, GSCs selectively modulate signaling pathways to regulate proliferation, infiltrative properties, survival, and metastasis, resulting in increased resistance to current therapies and tumor recurrence [21,22,23]. Studies in patient-derived GBM mouse models have shown prolonged survival by blocking self-renewal of GSCs [24], and characterizing stemness of GSCs while screening for effective drug combinations may be an effective strategy to improve treatment outcome in GBM [25].

Several studies highlight the need for a personalized approach to GBM treatment including functional precision medicine [16,26]. In this study, we present a multidisciplinary approach in different aspects of GBM treatment strategy. Human GBM tumors resected from patients cultured as 3D organoids were screened for differentially expressed genomic targets using next generation sequencing and susceptibility to specific inhibitors by testing against a library of 128 FDA-approved oncologic drugs using a high throughput approach. The capability to quickly (averaging within 6 weeks) and effectively screen genomic variabilities and a large number of effective compounds in a synchronized timeframe help generate a clinically useful prediction in a timely manner. In combination with markers of tumor stemness, we can highlight dynamic phenotypic changes as the tumor evolves during recurrences or due to treatment that may affect drug response. We sought to further address potential translational problems by testing the efficacy of selected drugs used in our in vitro assay with PDX models using immunocompromised NOG mice, with the overarching goal to validate our in vitro prediction model. All this was achieved in a timely fashion that fits into a clinically relevant time window that would be applicable to assist physicians to make an informed evidence-based decision, likely at the point just after the first tumor resection. The unique representative patient example in this manuscript presented initially with a primary grade IV GBM tumor and two subsequent local recurrences. Interestingly, the first recurrence was not preceded by any chemotherapy or radiation therapy, while the second recurrence was post chemotherapy (standard of care—temozolomide) and high-dose radiation. Assessment of tumors from a single patient mitigated variables associated with inter-patient idiosyncrasies. Thus, our study impresses the importance of a precision-based personalized approach and use of patient-derived 3D organoid tumor cultures to generate predictive models of treatment for GBM.

## 2. Materials and Methods

### 2.1. Tumor Processing

Resected tumors, de-identified under an IRB-approved protocol (1R01CA229737-01) at Boca Raton Regional Hospital (BRRH), MNI, FL, were delivered to UF Scripps in sterile 1X DPBS on wet ice within two hours of surgery. A typical resection ~1 cubic centimeter of solid tumor was disaggregated immediately on receipt in GBM media as described previously [27]. Briefly, mechanical disaggregation on ice using a surgical scalpel in a sterile hood separated the tumor into a predominantly single cell suspension. Cells and tumor debris resuspended in GBM serum-free media were centrifuged at 320× *g* for 5 min to form a pellet. Tumor cells were then filtered through a 70-micron cell strainer and centrifuged as before and later counted and re-suspended in GBM serum-free media. After red blood cell removal using Ficoll-paque, tumor cells, typically ~25 million, were plated in a T-25 flask, with 10 mL of GBM media, and incubated at 37 °C, 5% CO_2_, 95% humidity. The other half was placed in 5 mL of serum-free freezing media and divided into 5 vials of approximately 5 million cells per vial. These were stored in an LN2 freezer for future use as part of the flow cytometry, RNAseq analysis, and in vivo models. Cells in the culture were monitored over days, and typically 3D organoids were observed within a few days to a week after seeding. Tumor organoids were then expanded by collection and treatment with TryPLE to disaggregate the 3D organoids into single cell suspensions followed by reseeding at large volumes and expanded until we achieved enough spheres for HTS (~30–40 million cells total). This was typically achieved within a maximum period of 6 weeks.

### 2.2. FDA-Approved NCI Drug Library

A subset of the NCI-approved oncology drugs, 128 out of the current 179 total, were screened at the Scripps Research Institute Molecular Screening Center (SRIMSC) and reformatted into 1536-well source plates for automated robotic screening [16]. The reason for choosing only 128 was because that number fits uniquely and perfectly well on 1536-well source plates as 10 pt 3-fold serial dilutions. In addition, at the time when UF Scripps obtained that library from the NCI, there were only ~140 drugs. It was thus deemed most streamlined to test the 128 drugs used in this study (Supplemental Figure S1).

### 2.3. 3D Culture and 3D Viability Assay

The 3D cell viability assay was initially developed at UF Scripps in a 384-well format and then further miniaturized into a 1536-well format and performed as previously described with some changes (8,[27,28],29, 30). The 3D viability assay uses a detection reagent more adapted to organoids that features a tailored lysis buffer (Cell Titer-Glo 3D, Part# G9683, Promega, Madison, WI, USA). The assays were optimized by testing different variables, including cell number, aggregation time, incubation time, and time of drug addition. For all tumor samples, after growth was established per the methods described above, the organoids were disaggregated using TryPLE, the cells were placed in GBM media and counted, and 1250 cells/well in 5 µL culture media were seeded in 1536-well Greiner Bio-One flat-bottom cell-repellent plates (Part #789979, Greiner Bio-One, Monroe, NC, USA) followed by incubation for 24 h to allow cells to form 3D structures. Cells were then treated with drugs or vehicle (50 ηL or 10 ηL, 0.1% DMSO) [29]. Cell viability was assessed after 72 h incubation using Cell-Titer Glo 3D reagent according to the manufacturer’s instructions. Concentration response curves (CRC) and IC_50_ values of 3 or 5 pharmacological control compounds were used as the guide for assay optimization and drug screening. As a point of comparison, we also tested these cells using Corning organoid/spheroid plate technology (Part #3830, Corning Inc., Corning, NY, USA) [30]. The formation of a 3D structure was always confirmed by Hoechst staining followed by confocal imaging using an INCell Analyzer 6000 high-content reader, which are then used for Z-stack analysis as previously described [27].

### 2.4. Data Processing

A 10-point 3-fold serial dilution method was used to produce a concentration response curve against the patient-derived GBM tumor spheres. All data files obtained were uploaded into UF Scripps’ institutional database for individual plate quality control and hit selection. Assay plates were determined acceptable only if their Z’ was >0.5 [31].

Compound activity was normalized on a per-plate basis using the following equation: [32]

% inhibition = 100 × ((1 − (Test Well − Median High Control/Median Low Control − Median High Control))

*Test Well* refers to those wells with cells treated with test compounds. 

*High Control* is defined as wells containing medium only (100% inhibition), and

*Low Control* wells contain cells treated with DMSO only (0% inhibition). 

High and low controls were applied for assay quality evaluation in terms of Z’ [31]. Day to day assay response and stability was assessed using 5 pharmacological control compounds that we tested for their CRC and that were required to be within 3-fold of the expected IC_50_, on an experimental basis, for each tumor model. 

### 2.5. Concentration Response Assays

The 10-point, 3-fold serial dilutions of selected drugs were tested against GBM cancer-derived cells (BRRH-001, 001B, and 001C) in 3D format in triplicate starting from 5 µM nominal concentration. This same procedure was also done with the controls (doxorubicin, gemcitabine, SN-38, TMZ, and irinotecan) (Supplemental Figure S2). For each test compound, % inhibition was plotted against compound concentration. A four-parameter equation describing a sigmoidal dose-response curve was then fitted with an adjustable baseline using Assay Explorer software (Version 3.2, Symyx Technologies, Santa Clara, CA, USA). The reported IC_50_ values were generated from fitted curves by solving for the X-intercept value at the 50% inhibition level of the Y-intercept value. In cases where the highest concentration tested (i.e., 5 µM) did not result in greater than 50% cytotoxicity, the IC_50_ was deemed as greater than 5 µM. 

### 2.6. In Vivo Profiling

Mice: All animal studies were conducted with institutional oversight under an IACUC-approved animal care and use protocol (ACUP) at Certis Oncology Solutions using adult female NOG mice (Taconic, Germantown, NY, USA).

Preparation of Cells for Xenotransplantation:

Cells were grown and subcultured in GBM media as described previously [28]. On the day of implantation, tumor spheres were dissociated into a single cell suspension using Accumax Cell Dissociation Solution (Innovative Cell Technologies, Novi, MI, USA. #AM105) according to standard manufacturer recommendations. Cell numbers and viability were quantified using the Countess II Automated Cell Counter (Invitrogen, Waltham, MA, USA). Cells were resuspended in either GBM media (intracranial implants) or PBS:Matrigel (50:50) (subcutaneous implants) (Corning, Tewksbury, MA, USA. #356237) at the appropriate density for in vivo implantation. Cell suspensions were kept on ice until injection. 

### 2.7. Intracranial Orthotopic Xenotransplantation

Stereotactic intracranial implants were conducted in a manner similar to previous methods with minor modifications [33]. Female NOG mice (aged 6–8 weeks) were anesthetized via isoflurane induction and checked for appropriate surgical plane anesthesia by rear toe pinch. The surgical site was shaved and prepared using aseptic technique to scrub the skin, with 3 rounds of alternating surgical scrub and alcohol. Artificial tears were applied to the eyes to prevent dryness. A 3–4 mm rostral/caudal incision was made through the skin of the head to expose the animal’s skull before transferring and securing the animal to the stereotactic platform. A microdrill (Stoelting, Wood Dale, IL, USA. #58610) was used to drill through the skull at the injection coordinates, X = 1.5 mm and Y = 0.5 mm from Bregma. Once the infusion needle (Hamilton, Reno, NV, USA. #CAL80383) was loaded and primed with cells, it was repositioned at the injection coordinates. The needle was lowered to *z*-axis = −3.5 mm for one minute, then raised back up to *z*-axis = −3.0 to begin infusion. A 4 μL injection volume containing 3.0 × 10^5^ viable cells was injected via infusion pump at a rate of 2 μL/min (Harvard Apparatus Pump 11 Elite, Holliston, MA, USA). The needle was left in place for 1 additional minute following cell injection before being slowly retracted. Bone wax was applied to the skull to plug the injection site, and interrupted sutures were used to close and secure the incision. Post-operative analgesic was administered. Intracranial tumor growth was monitored using T2 weighted (T2W) contrast on the M3 Compact MRI (1 Tesla) (Aspect Imaging, Nashville, TN, USA). Intracranial tumor volumes were quantified using VivoQuant software (Version 2021, Invicro, Needham, MA, USA).

### 2.8. Subcutaneous Xenotransplantation and Pharmacology

#### 2.8.1. Subcutaneous Model Build

Animals were shaved and surgically prepped using 70% isopropyl alcohol and surgical scrub. Subcutaneous xenografts were established by injecting a 100 μL volume of Matrigel:PBS (50:50) containing 3.0 × 10^5^ viable cells into the right flank of adult female NOG mice. Tumor volumetric measurements were conducted twice weekly using standard caliper procedures. Body weights were also measured twice weekly throughout the length of the studies. 

#### 2.8.2. Pharmacology

A total of 60 female NOG mice (aged 5–8 weeks) were implanted by subcutaneous injection as described above. Tumor volumes were monitored twice weekly by caliper measurement until a minimum of 40 tumors reached a combined average volume of 90–100 mm^3^ for randomization. Mice were randomized using the matched distribution method into 4 groups containing 5 animals per group for initiation of pharmacologic dosing). Vehicle (Control), vemurafenib (10 mg/kg) (MedChem Express, Monmouth Junction, NJ, USA. #HY-12057), cobimetinib (5 mg/kg) (MedChem Express, Monmouth Junction, NJ, USA. #HY-13064), and combination group treated with cobimetinib (5 mg/kg) and vemurafenib (10 mg/kg) were formulated using stepwise addition of the following solvents: 10% DMSO, 40% PEG300, 5% Tween-80, and 45% saline. Vehicle, vemurafenib, and cobimetinib and cobimetinib-vemurafenib combination were dosed once daily by oral gavage (QD, PO)). Animals were dosed QD for the first 20 consecutive days and then placed on a QDx5/week dosing schedule until Day 48 or another appropriate humane endpoint. Tumor volumes and body weights were assessed twice weekly for the duration of the study. Upon reaching a humane study endpoint, tumors were harvested and processed for histopathological assessment by standard hematoxylin and eosin (H and E) staining techniques and immunohistochemical analysis.

#### 2.8.3. Flow Cytometry and Phenotypic Stemness Characterization

Spectral flow cytometric analysis was performed using the Cytek Aurora flow cytometer (Cytek Biosciences, Fremont, CA, USA). Cells were harvested, dissociated into single cell suspensions using Accumax Cell Dissociation Solution, and stained for the following stemness markers using fluorochrome-conjugated monoclonal antibodies (CD133, CD15, CD44, CD36, Nestin, CD49f) as follows. Single cell suspensions were transferred to a V-bottom 96-well plate for flow cytometry staining and washed using FACS Buffer (PBS + 2% FBS + 2 mM EDTA). Cells were first stained using zombie aqua viability dye in PBS for 10 min. at 4 °C in the dark. Cells were then washed with FACS Buffer and resuspended in an antibody cocktail containing anti-CD133-PE, anti-CD15 PECy7, anti-CD44-BV757, anti-CD36 AF700, anti-Nestin AF488, anti-CD49f APCFire750, and anti-mouse H2Kd-BV421. Cells were incubated for 20 min. at 4 °C in this antibody cocktail before being washed, fixed, and analyzed on the Cytek Aurora spectral flow cytometer. Analysis of stemness marker expression using overlays or T-distributed Stochastic Neighbor Embedding (t-SNE) was performed on the live, human cell population (mH2Kd-, zombie aqua) using FlowJo analysis software, Version 10.8.1 (BD Biosciences, San Jose, CA, USA).

## 3. Results

### 3.1. In Vitro Cytotoxic Effects of Cobimetinib, Vemurafenib, and Their Synergistic Combination to 3D GBM Organoids

**Figure 1:**
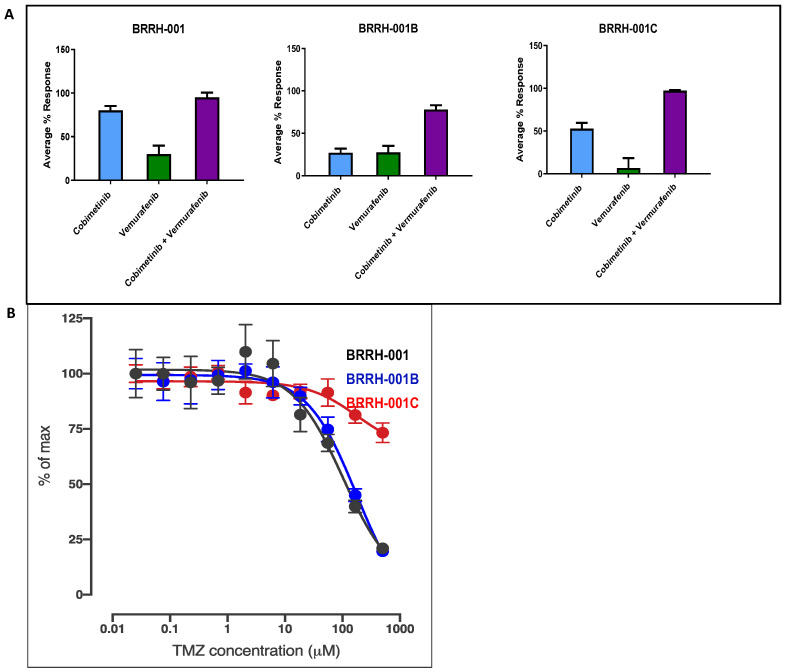
In vitro high-throughput drug screening (HTS) using human patient-derived GBM spheroids. Variable drug sensitivity of patient-derived GBM spheroids cultured between primary and recurrent tumors excised from the same patient. (**A**) In vitro drug response, derived from viability assay of patient-derived GBM spheroids treated with sub IC-50 concentration (20 μM) of cobimetinib, vemurafenib, or a combination of both shown as avg % cytotoxicity or (**B**) standard of care temozolamide for 72 h shown as % of max growth. BRRH-001 is the primary tumor, while BRRH-001B and BRRH-001C are subsequent recurrences. Panel (**A**) is a representative drug combination from 128 NCI-approved compounds tested using HTS screening and 8 selected drug combinations. (N = 6 for single drug test, N = 9 for combination drug test, run in triplicate. All data are mean ± SD.)

**Figure 2:**
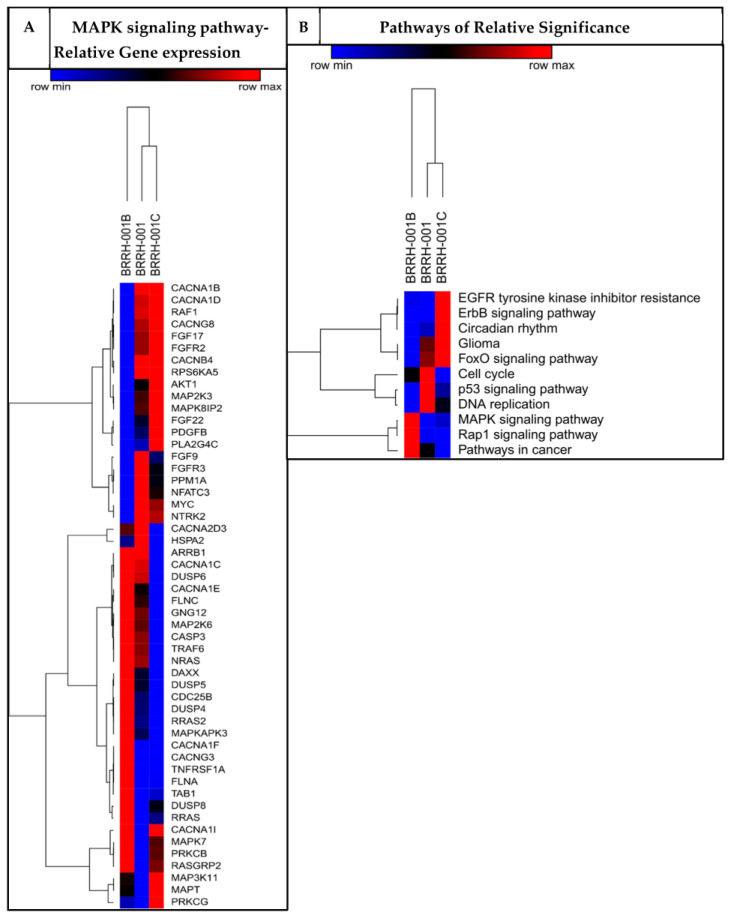
RNAseq analysis of patient-derived GBM organoids. RNAseq analysis of the patient-derived GBM tumor organoids cultured from primary and recurrent GBM tumors resected from the same patient. BRRH-001 is the primary tumor, while BRRH-001B and BRRH-001C are subsequent recurrences. Comparative analysis of the patient-derived GBM RNA transcripts to the healthy brains subset of the GTEx database (an NIH reference dataset). (**A**) shows the breakdown of the significantly modulated genes in the MAPK pathway (see Supplemental Figures for the genes of other enriched pathways), and (**B**) shows a heat map of all the significantly enriched pathways. Expression in the heatmap is in the order of log P.

We generated GBM 3D organoids from a surgically resected patient-derived primary tumor. The average time frame for establishing sufficient 3D organoids for all in vitro testing was 6 weeks, while most tumors were established relatively earlier. We used a high-throughput phenotypic assay (HTS) to rapidly evaluate cytotoxicity of 128 oncologic compounds from an NCI-approved drug set and several synergistic combinations at sub IC50 concentrations against patient-derived GBM organoids. Following the in vitro analysis (Figure 1), we obtained RNAseq data and applied additional insight from this analysis (Figure 2, Supplemental Figure S2) to further assess sensitivity of patient-derived GBM organoids to several kinase inhibitors, including synergistic effects of the cobimetinib and vemurafenib combination. We found variable sensitivity for 20 uM cobimetinib and vemurafenib monotherapy in the primary GBM tumor (BRRH-001A) and subsequent recurrences (BRRH-001B and BRRH-001C) (Figure 1A). For cobimetinib, BRRH-001B showed decreased sensitivity by almost 75% compared to the primary tumor, while BRRH-001C was 25% more sensitive than BRRH-001B, indicating developing drug resistance in recurring tumors. Comparatively, vemurafenib showed lower toxicity (less than 30%) in the BRRH-001A and BRRH-001B and even lower effect in the second recurrence, BRRH-001C. However, the combination of cobimetinib and vemurafenib showed a consistently synergistic effect (~100% toxicity) in the primary tumor and all the recurrences. There was a slight decrease in BRRH-001B (Figure 1A).

We also evaluated the sensitivity of these patient-derived GBM organoids to the standard of care temozolomide. At 800 uM (within the IC-50 range), we observed decreased response in BRRH-001C with loss of almost 65% sensitivity by the second recurrence (BRRH-001C), indicating treatment resistance (Figure 1B).

### 3.2. Patient-Derived 3D Organoids Show Differentially Enriched Pathways of Growth and Development

To supplement the functional HTS assessing drug sensitivity, we applied RNAseq of the GBM organoids retrospectively as a predictive genetic and molecular analysis. We evaluated modulation of growth and signaling pathways in the GBM organoids using comparative analysis with the Genotype-Tissue Expression (GTEx) database of global mRNA expression in healthy human brains. We identified several enriched pathways, including MAPK signaling, EGFR tyrosine kinase, ErbB signaling, cell cycle, P53 signaling, and Rap1signaling (Figure 2A, Supplemental Figure S4) in the GBM organoids compared to healthy brains. While pathways involved in DNA replication, cell cycle, and p53 signaling are dysregulated in all three organoids, the *MAPK* and *Rap1* signaling pathways are enriched in the first recurrence, but the *EGFR, ErbB*, and *FoxO* signaling pathways are more dysregulated in the second recurrence. These findings support the investigation of inhibitors of these pathways as potential targets against drug resistance and anti-tumor efficacy. Combining information from our primary in vitro screening against a 128-compounds oncology dataset using our previously validated high-throughput screening method (Supplemental Figure S1) [8,34], we identified several targets, and specifically the dysregulated genes within the *MAPK* pathway (Figure 2B) showed susceptibility to specific drugs with possible synergistic combinations. Thus, we focused on several effective kinase inhibitors of the MAPK pathway, including cobimetinib, vemurafenib, trametinib, and dabrafenib (Figure 1A, Supplemental Figure S3).

### 3.3. Orthotopic or Subcutaneously Implanted GBM Organoids Have Similar Growth Profile in Mice

**Figure 3:**
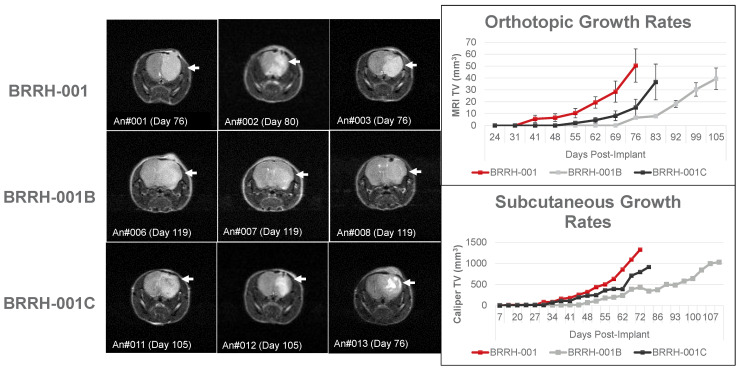
Validation of orthotopic vs. subcutaneous in vivo tumor growth model in mice. Comparing variable tumor growth in mice without any treatment using patient-derived GBM spheroids from primary or recurrent tumors excised from the same patient. These spheroids were implanted orthotopically (OT) into the brain or subcutaneously (SQ) into the flank. Brain orthotopic measurements were done using MRI and SQ measurements with calipers. Tumor volume was monitored for orthotopic and SQ over a period of greater than 100 days. White arrows in the MRI indicate tumor growth. (OT model n = 13, SQ model n = 1; all data represent mean ± SEM).

To validate different in vivo mouse tumor growth models, an equal amount of patient-derived GBM organoids (3 × 10^5^ viable cells/mouse) were implanted orthotopically (OT) into the brain or subcutaneously (SQ) into the flank of adult female NOG mice. Tumor volume monitored over a period greater than 100 days (average of 76 days in both models) showed similar growth kinetics (Figure 3). OT tumors were measured by T2 weighted (T2W) contrast using a compact MRI, while SQ tumors were measured using standard calipers. Tumor volume measurement in both models had varying scales but showed similar kinetics and maximum tumor volume among the GBM organoids from the primary tumor and subsequent recurrences. With respect to tumor growth kinetics, the primary tumor (BRRH-001A) had the highest growth rate, and the first recurrence (BRRH-001B) had the lowest. Interestingly, the second recurrence (BRRH-001C) seemed to gain back some tumorigenic potential lost by BRRH-001B.

### 3.4. In Vivo Validation for Toxicity of Vemurafenib and Cobimetinib and Their Combination to Patient-Derived GBM Organoids

**Figure 4:**
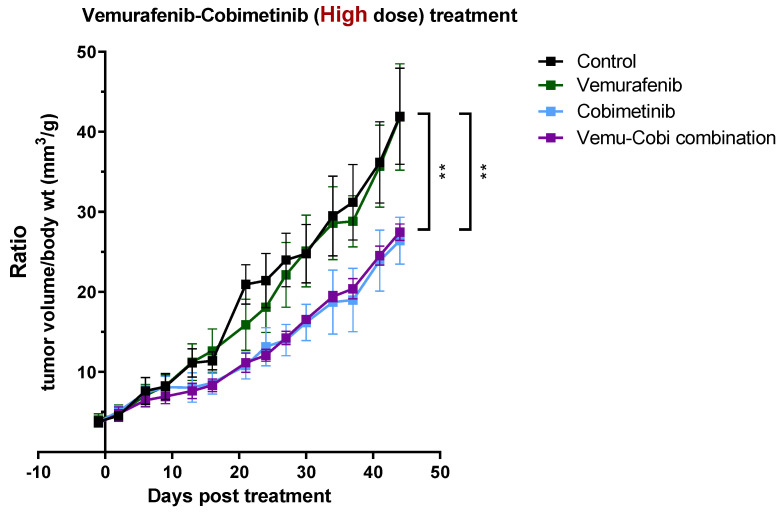
In vivo antitumor efficacy of synergistic drug combination in mice. Mice subcutaneously implanted with patient-derived spheroids cultured from a primary tumor (BRRH-001) were treated with control placebo or cobimetinib or vemurafenib or a combination of both. Tumor volume and body weights were monitored at regular intervals starting 48 h prior to treatment. (N = 4 mice in each group, all data are mean ± SEM; 2-way ANOVA and multiple comparison against control group with Bonferroni correction; ** *p* < 0.01).

Since the primary tumor, BRRH-001A, showed the most aggressive tumor growth kinetics, we subcutaneously implanted 3 × 10^5^ viable GBM organoids into the flank of female NOG mice (aged 5–8 weeks). Mice were randomized at an average tumor volume of 90–100 mm^3^ to receive the drug diluent (control) or cobimetinib (5 mg/kg) or vemurafenib (10 mg/kg) or a combination of both (see details of dosing schedule in Section 2). Tumor volume and body weights were monitored twice weekly at regular intervals starting 48 h prior to treatment for up to 48 days or another humane end point. Cobimetinib treatment in mice showed statistically significant inhibition, ~40% reduction in tumor growth when compared to mice in the control and vemurafenib treatment groups without any difference in toxicity. Tumor volumes compared were body weight matched. (An amount of 40 mm^3^/g in control vs. 25 mm^3^/g in cobimetinib group, n = 4/group, *p* < 0.01) (Figure 4). Interestingly, vemurafenib treatment by itself did not show any effect compared to the control group, whereas the combination group matched the efficacy of cobimetinib monotherapy, which was concordant with the in vitro findings (Figure 1A). As further validation of these results, we added the normality testing to justify the use of ANOVA as Supplemental Figure S6. In addition to presenting the tumor growth plot as a ratio of weight vs. tumor size to address the concern of drug toxicity, we have also added the body weight vs. time plot as Supplemental Figure S5.

### 3.5. Patient-Derived 3D GBM Organoids from a Primary and Recurrent Tumor Show a Heterogeneous “Stemness” Signature

We generated 3D GBM organoids (details in Section 2) from a single patient presenting with a primary GBM tumor and multiple recurrences. Using flow cytometry, we assessed the expression of well-known stem cell surface markers including CD44, nestin, CD133, and CD49f on the 3D organoids.

**Figure 5:**
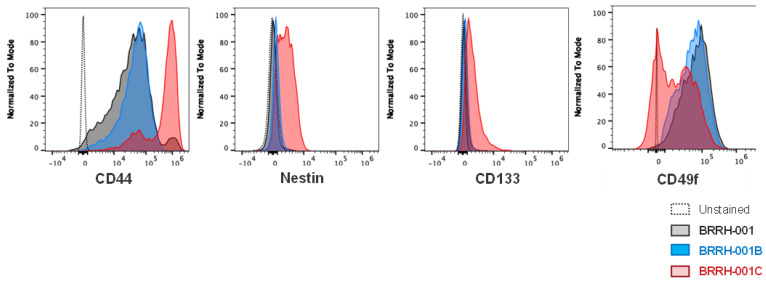
Heterogeneous expression of stem cell markers in patient-derived GBM spheroids cultured in vitro. Expression of neuronal stem cell markers in patient-derived GBM spheroids cultured from primary or recurrent tumor from the same patient was assessed using flow cytometry. Level of expression of neuronal markers of stemness is assessed as Mean Fluorescence Intensity (MFI) and compared as histogram overlays of the different GBM spheroids. BRRH-001 is the primary tumor, while BRRH-001B and BRRH-001C are subsequent recurrences.

We observed similar expression profiles of all these markers in the primary tumor (BRRH-001) and the first recurrence (BRRH-001B), while the second recurrence (BRRH-001C) demonstrated a comparatively different profile. We observed upregulation of CD44, nestin, and CD133 in all the samples, with marked increase in BRRH-001C compared to BRRH-001 and BRRH-001B. For CD49f, we observed a bi-modal distribution of expression in BRRH-001C, with a significant proportion of the population with downregulated CD49f (Figure 5).

**Figure 6:**
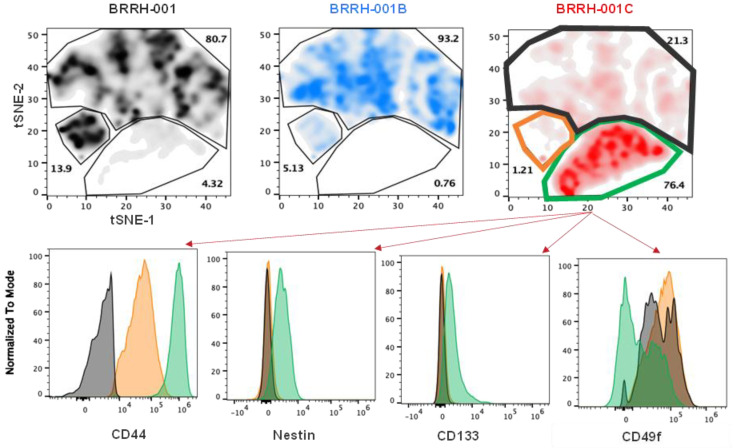
*t*-SNE plot analysis of stem cell markers in patient-derived GBM spheroids cultured in vitro. *t*-SNE analysis of the neuronal markers of stemness identifies changes in the distribution of these markers between GBM spheroids cultured from primary or recurrent tumors. Representative histogram overlays of the different neuronal markers from the recurrent tumor, BRRH-001C, shows a variable distribution of these markers. The histogram plot colors match the t-SNE plot overlay of BRRH-001C.

Additionally, using t-SNE plot analysis, we also evaluated population-based differences in all three GBM recurrences based on the expression of these markers. In BRRH-001C, we observe a significant increase in the clustering of a population (76.4% vs. 13.9% in BRRH-001 and 0.76% in BRRH-001B) expressing CD44, nestin, and CD133 (Figure 6). This population cluster showed a bimodal distribution for CD49f (Figure 6, green bars). Conversely, there was a significant decrease in the BRRH-001C, more than 60%, in the population cluster exclusively CD49f positive (21.3% vs. 80.7% in BRRH-001 and 93.2% in BRRH-001B) (Figure 6, black bars). A similar decrease was also observed in a cluster, with more than 90% in a cluster positive for both CD44 and CD49f (1.21% vs. 13.9% in BRRH-001 and 5.13% in BRRH-001B). Interestingly, this population did not show a bi-modal distribution for CD49f (Figure 6, orange bars).

## 4. Discussion

In this study, we demonstrate feasibility of an integrated precision medicine approach combining genetic and molecular analysis of the tumor with functional in vitro assays and in vivo tumor growth kinetics to guide an effective treatment strategy for GBM. Such personalized and comprehensive approaches to solid tumors have been limited to date and are imperative to combat treatment resistances and failures due to the highly heterogeneous, resistive, and invasive phenotype of GBM tumors. Our 3D culture technology, when combined with stronger genotypic and phenotypic concordance with parent-tumor and HTS, facilitates rapid identification of effective compounds or drugs [27,34,35,36,37]. There is an urgent need to establish similar systems for GBM tumors, considering that current options for GBM treatment only provide limited benefits [38]. Historically, treatment strategies have focused on expression of key protein targets or targetable mutations, such as HER2 targeted therapy in breast cancer or EGFR targeting in lung cancer. However, only a small minority of individuals (38% or less) match a genomic targeted therapy, and even a smaller proportion (5–7%) actually show a positive response [39,40,41,42]. A possible reason might be overestimation of the impact of these genomic drivers on cancer phenotypes, since several studies indicate a significant role of non-genetic determinants and epigenetic mechanisms in acquired resistance observed in many cancers [43,44].

In our study, we could identify significant sensitivity of the patient-derived 3D organoids to several modulators of MAPK and MEK signaling pathways, among several others, by utilizing a functional high-throughput screening (HTS) against an FDA-approved NCI collection of 128 drugs (Figure 1) (Supplementary Figures S1 and S3). Additionally, we used next-gen RNAseq analysis of patient-derived 3D GBM organoids to supplement the functional phenotype assay and identify potential genomic targets along with several differentially enriched molecular pathways that could be exploited by targeted drug therapy (Figure 2) [45]. We could thus identify single drug targets as well as possible synergistic combinations of drugs specific for a patient by combining the genomic analysis with a functional HTS. In the same patient, we observed that the primary tumor and two recurrences, each from a different point in the treatment course, had varying sensitivity to drugs, and even observed resistance to the standard of care temozolomide in the last recurrence (Figure 1B). The patient had not received any treatment before/after the primary tumor resection but received chemotherapy and radiation after the first recurrence was resected. This observation shows that tumors evolve over time, even without treatment, highlighting the impact of tumor heterogeneity and clonal selection on developing treatment resistance. Historically, only with rare exceptions have single anti-metabolites been able to achieve sustained responses, but rather using a combination of such drugs has really made it possible to achieve sustained responses and cures for most cancers. Using a synchronized genomic approach with an integrative precision medicine approach in a more relevant 3D tumor culture, combined with in vivo PDX mouse models using tissue derived from affected individuals, one could possibly assess and identify effective drugs and combinations that may be the answer to overcoming vulnerabilities in the genomic-only approach [16].

Use of pre-clinical models such as patient-derived xenografts (PDX) and patient-derived organoids (PDO) to better recapitulate patient tumor heterogeneity may be a refined solution to improve translation of drug sensitivity and improve therapeutic outcomes. For example, the FORESEE Clinical Trial for Her2 negative metastatic breast cancer is utilizing patient-derived primary and metastatic tumor tissue for genomic sequencing and developing an organoid model for drug screening, thus aiding the physician to select an optimal therapy (ClinicalTrials.gov: NCT04450706). Recent studies have clearly implicated the local tumor microenvironment in not just governing tumor biology and growth but also affecting tumor response to drugs [17]. We utilized NGO mouse models for in vivo translational validation of the synergistic drug predictions based on the in vitro 3D culture. We established orthotopic and subcutaneous tumor models from patient-derived 3D organoids used in the HTS in vitro prediction model and compared growth rates between each model (Figure 3). Observing similar growth patterns, we tested the efficacy of the predicated drug combinations on tumor growth in the subcutaneous mouse models. Understandably, subcutaneous mouse tumor models have translational limitations to orthotopic models; however cost and technical resources, as well as time to establish tumors in mice, are important considerations when including in vivo validation of drug efficiencies in a functional personalized precision prediction workflow. Primary tumor (BRRH001) growth studies in mice indicated concordance with the in vitro predictions of drug efficiencies and no significant difference in toxicities (Figure 4). There was no synergy with the drug combination in the animal model, but the anti-tumor effect of cobimetinib monotherapy, which matched the combination group effect, and the lack of effect with vemurafenib monotherapy were in concordance with our observation from the in vitro screening.

Assessing stemness of the GBM tumor is expected to have critical implications for tumor drug response. Several mouse studies using human glioma xenografts and primary patient glioblastoma specimens have revealed tumor stemness as a determinant in development of treatment resistance, and targeting specific tumor stem cells might improve survival [21,24,46]. We evaluated the stem cell signature in these patient-derived organoids and found significantly varying levels of stemness, especially in levels of CD44, nestin, and CD133 in our representative example patient tumor after receiving chemotherapy and radiation treatment (Figure 5 and Figure 6). This variation does correlate with the drug response and resistance indicated by BRRH-001C in the in vitro and in vivo models. This unique examination of the tumor over time helped us compare the molecular evolution of the tumor phenotype within a single individual with recurrence and standard chemotherapy and radiation, thus highlighting the need to evaluate tumor molecular phenotypes as the tumor progresses.

## 5. Conclusions

Our model reflects a multidisciplinary approach with strong coordination between neurosurgical care at the bedside and the discovery and testing arm. In summary, we show that tumor phenotype (stemness) may evolve with or without chemotherapy and radiation treatment. An integrative drug response prediction uses genomic, in vitro, and in vivo combination and patient-derived 3D in vitro models and PDX in vivo models as more accurate predictors of drug response. Thus, we believe that this novel functional personalized precision workflow has the potential to be rapidly implemented in the clinic setting, since it has the benefit of having a predictive capacity for identifying effective and relevant targets personalized for each individual patient and further validated using in vivo PDX mouse models completed in a clinically relevant timeframe. Moreover, it is possible to scale up this process further to include additional drugs that have shown promise in the clinical trial setups and are yet to be approved by the FDA for GBM treatment.

## Data Availability

The data presented in this study are available in this article (and Supplementary Material). Any additional information needed can be obtained by contacting the corresponding author.

## Supplementary Materials

The following supporting information can be downloaded at https://www.mdpi.com/article/10.3390/cancers15133289/s1: Figure S1: 128 drugs used in this study; Figure S2: Controls used for dose-response test of the tumor organoids against the NCI library (128 compounds); Figure S3: Control for synergy combinations (Dabrafenib and Trametinib); Figure S4: KEGG enrichment P-values and Volcano plots showing overview of differential expression of all the genes tested by RNAseq analysis; Figure S5: Body weight as a measure of toxicity during tumor growth and treatment; Figure S6: Normality testing of tumor growth data.
