# Supplementary material for: In Vitro and In Vivo Drug-Response Profiling Using Patient-Derived High-Grade Glioma
Source: Cancers (Basel). 2023 Jun 22;15(13):3289. doi: 10.3390/cancers15133289 (PMC10339991; doi:10.3390/cancers15133289)
Supplement: Supplementary file 1 [file cancers-15-03289-s001.zip › cancers-2408109-supplementary.pdf]

Supplemental figures

Figure S1 128 drugs used in this study

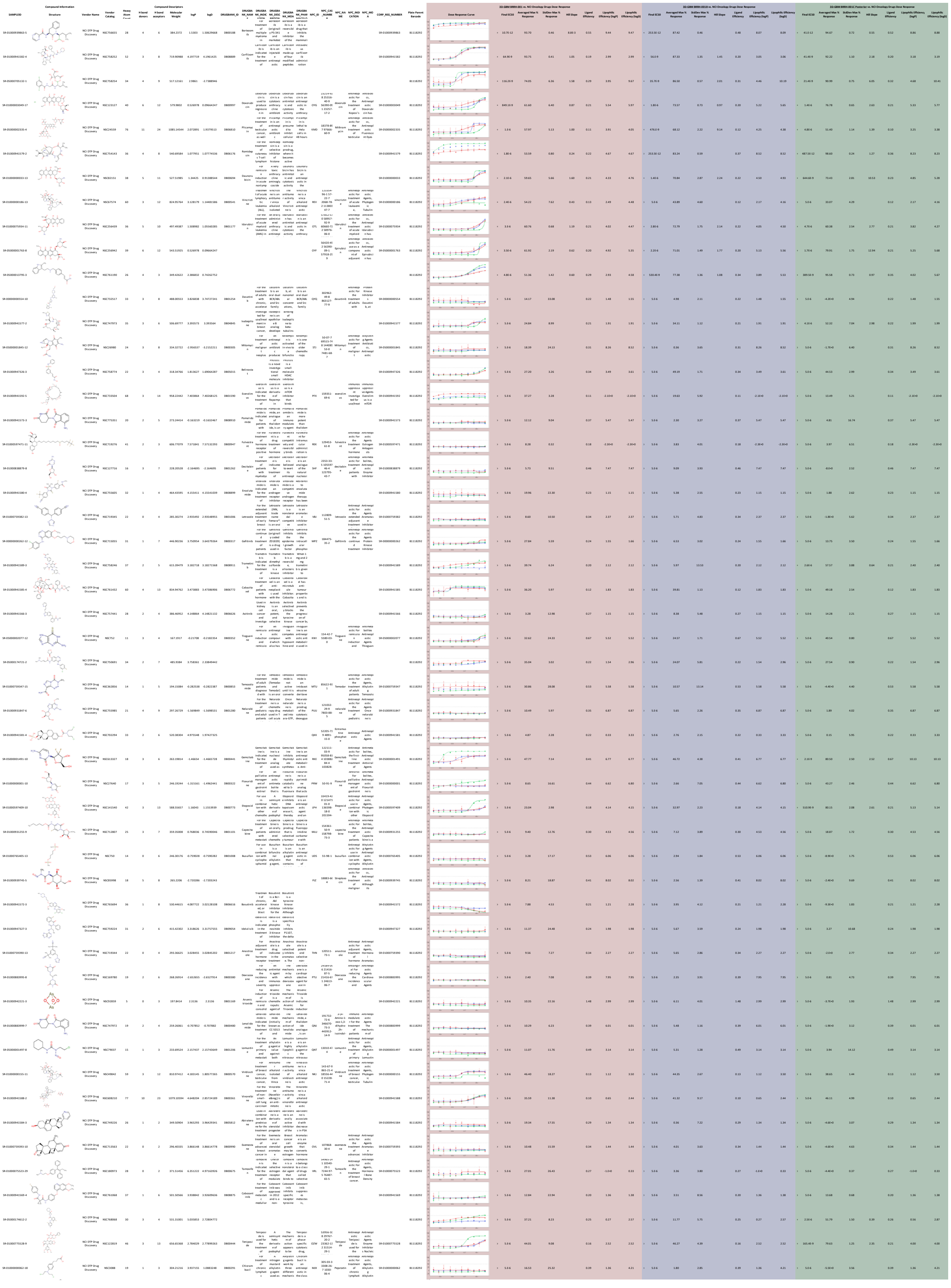

SR-01000941591-2	NCI DTP Drug Discovery	NSC226080	65	3	13	914.17186	7.45084	7.4496567	DB00877	Sirolimus	For the prophyla xis of organ rejection in	A macrolid e compoun d obtained	Sirolimus inhibits T lymphocy te activatio n and	Sirolimus , a macrocyc lic lactone produced						SR-01000941591	B1118292
SR-05000000373-14	NCI DTP Drug Discovery	NSC701852	19	3	3	264.3202	2.003969	1.99074056	DB02546	Vorinostat	For the treatment of cutaneou s manifest	Vorinost at (rINN) or suberoyl anilide hydroxa	Vorinost at inhibits the enzymati c activity		KWY	149647-78-9	vorinostat	Antineopl astic For the treatment of cutaneou	Anti-Inflamma tory Agents, Non-Steroidal	SR-05000000373	B1118292
SR-01000947328-3	NCI DTP Drug Discovery	NSC761910	33	1	6	440.49705	3.630226	3.57004032	DB09053	Ibrutinib	Ibrutinib was approved by the FDA for the	Ibrutinib is a small molecule anti-cancer	Ibrutinib is an inhibitor of Bruton's tyrosine	In patients with recurrent B-cell lymphom						SR-01000947328	B1118292
SR-05000001855-9	NCI DTP Drug Discovery	NSC6396	11	0	1	189.21834	-1.027381	-1.0273805	DB04572	Thiotepa	ThioTEPA is used a as condition ing treatment	N,N'N'-triethyler ethiopho sphorami de (ThioTEP	The alkyl group is attached to the guanine base of	The unstable nitrogen-carbon groups alkylate	MFC	52-24-4	Thiotepa	Antineopl astic ThioTEPA is used a as condition	Antineopl astic Agents, Alkylatin g Myeloabl	SR-05000001855	B1118292
SR-01000941575-5	NCI DTP Drug Discovery	NSC756645	30	2	5	450.33668	3.574117	0.95444061	DB08865	Crizotinib	Crizotini b is used for the treatment of locally advanced	Crizotini b an inhibitor of receptor tyrosine	Crizotini b is a tyrosine kinase receptor inhibitor.							SR-01000941575	B1118292
SR-01000930807-4	NCI DTP Drug Discovery	NSC34462	15	2	5	252.09784	2.152872	2.15286994	DB00791	Uracil mustard	Used for its antineopl astic propertie s.	Nitrogen mustard derivativ e of uracil. It is a	After activatio n, it binds preferent ially to	Uracil Mustard selectivel y inhibits the synthesis	TIF	66-75-1	Uramustine	Antineopl astic Used for its antineopl astic	Antineopl astic Agents, Alkylatin g After activatio	SR-01000930807	B1118292
SR-00000000462-6	NCI DTP Drug Discovery	NSC760766	30	1	6	475.35396	4.542965	2.81382799	DB05294	Vandetanib	Vandetan ib is currently approved as an alternati	Vandetan ib is an oral once daily kinase inhibitor	ZD-6474 is a potent and selective inhibitor	Mean IC50 of approxim ately 2.1 ?g/mL.						SR-00000000462	B1118292
SR-01000941571-6	NCI DTP Drug Discovery	NSC763932	33	3	4	482.81541	4.486032	4.48571527	DB08896	Regorafenib	Regorafe nib is indicated for the treatment of	Regorafe nib is an orally-administ ered inhibitor	Regorafe nib is a small molecule inhibitor of							SR-01000941571	B1118292
SR-05000001983-6	NCI DTP Drug Discovery	NSC1390	10	2	3	136.11146	-0.881783	-0.8955999							QPE	315-30-0 17795-21 0 184856 42-6 22767-92 6 39464-	allopurinol	Antiurolit hic For the treatment of hyperuric	Antimeta bolites Enzyme Inhibitor s Free Radical	SR-05000001983	B1118292
SR-05000174675-2	NCI DTP Drug Discovery	NSC616348	43	1	8	586.67801	2.776473	0.71578708													B1118292
SR-01000947686-3	NCI DTP Drug Discovery	NSC758247	33	2	8	447.53276	2.770733	1.29655424	DB09073											SR-01000947686	B1118292
SR-01000000239-30	NCI DTP Drug Discovery	NSC122758	22	1	2	300.43512	5.014368	2.64062965	DB00755	Tretinoin	For the induction of remissio n in	Tretinoin also known as all-trans-retinoic acid	Tretinoin binds to alpha, beta, and gamma retinoic	Tretinoin also known as all-<i>trans</i>-	PEP	5300-03-8 302-79-4 4759-48-2	Tretinoin	Antineopl astic For topical treatment of cutaneou	Antineopl astic Agents Alitretino in binds to and	SR-01000000239	B1118292
SR-05000001945-9	NCI DTP Drug Discovery	NSC312887	24	5	11	365.21168	-1.950066	-5.1241034							MTZ	75607-67 9 71261-44-4	Fludarabine	Antineopl astic For the treatment of adult patients	Antimeta bolites, Antineopl astic Immunos uppressi	SR-05000001945	B1118292
SR-05000002022-9	NCI DTP Drug Discovery	NSC109724	14	1	2	261.08596	0.096548	0.09654713	DB01181	Ifosfamide	Used as a compone nt of various chemothe rapeutic	Ifosfamid e is a chemothe rapeutic agent chemical	The exact mechanis m of ifosfamid e has not been	Ifosfamid e requires activatio n by microso	SEV	3778-73-2 66849-34-1 66849-33 0 36341-88-5	Ifosfamide	Antineopl astic Used as a compone nt of various	Antineopl astic Agents, Alkylatin g Immunos	SR-05000002022	B1118292
SR-01000075751-15	NCI DTP Drug Discovery	NSC38721	18	0	0	320.0412	6.110365	6.11036479	DB00648	Mitotane	For treatment of inoperab le adrenoco	A derivativ e of the insectici de dichloro	Its biochemi cal mechanis m of action is	Mitotane is an oral chemothe rapeutic agent indicated	NGN	53-19-0	Mitotane	Antineopl astic For treatment of inoperab le	Antineopl astic Agents, Hormona l Its biochemi	SR-01000075751	B1118292
SR-01000075662-21	NCI DTP Drug Discovery	NSC102816	17	4	8	244.20468	-3.065333	-3.065336	DB00928	Azacitidine	For treatment of patients with the following	A pyrimidi ne nucleosi de analogue	Azacitidi ne (5-azacytidi ne) is a chemical analogue	Azacitidi ne is believed to exert its antineopl	MGP	320-67-2 65886-71 7	azacitidine	Antineopl astic For treatment of patients with the	Antimeta bolites, Antineopl astic Enzyme Inhibitor	SR-01000075662	B1118292
SR-01000939639-7	NCI DTP Drug Discovery	NSC737754	31	2	7	437.51801	3.548707	3.547946	DB06589	Pazopanib	Treatmen t of advanced renal cell cancer and	Pazopani b is a small molecule inhibitor of	Pazopani b is a second-generatio n multitarg	Pazopani b is a synthetic indazolyl pyrimidi ne and	MUB	790713-33-6 444731-52-6 635702-64-6	Pazopanib	Antineopl astic	Antineopl astic Agents	SR-01000939639	B1118292
SR-01000837528-12	NCI DTP Drug Discovery	NSC719627	26	1	3	381.37216	4.009442	4.00924962	DB00482	Celecoxib	For relief and managem ent of osteoarth ritis (OA),	Celecoxib is a non-steroidal anti-inflamma tory drug	The mechanis m of action of celecoxib is	Celecoxib , a selective cyclooxy genase-2 (COX-2)	OBU	169590-42-5 184007-95-2 194044-54-7	celecoxib	Antiinfla mmatory For relief and managem ent of	Anti-Inflamma tory Agents, Non-Steroidal	SR-01000837528	B1118292
SR-05000174566-2	NCI DTP Drug Discovery	NSC766270	61	3	11	868.4392	6.760729	6.8847725													B1118292
SR-01000075736-14	NCI DTP Drug Discovery	NSC409962	12	1	3	214.04986	1.022656	1.02264576	DB00262	Carmustine	For the treatment of brain tumors, multiple myeloma,	A cell-cycle phase nonspeci fic alkylatin	Carmusti ne causes cross-links in DNA and	Carmusti ne is one of the nitrosour eas indicated	LYG	154-93-8 59099-88 6	Carmustine	Antineopl astic For the treatment of brain tumors,	Antineopl astic Agents, Alkylatin g Carmusti	SR-01000075736	B1118292
SR-01000763561-14	NCI DTP Drug Discovery	NSC743414	37	2	7	493.60274	4.377673	3.45150696	DB00619	Imatinib	For the treatment of Philadelp hia chromos	Imatinib is a small molecule kinase inhibitor	Imatinib mesylate is a protein-tyrosine kinase	Imatinib is an antineopl astic agent used to	KLZ	152459-95-5 220127-57-1	Imatinib	Antineopl astic For the treatment Philadelp hia	Antineopl astic Agents Protein Kinase Inhibitor	SR-01000763561	B1118292
SR-05000001667-5	NCI DTP Drug Discovery	NSC8806	19	2	4	305.20021	0.252196	0.2493237							URY	148-82-3 531-76-0 4213-32-5 3223-07-2 13469-52	Melphalan	Antineopl astic For the palliative treatment of	Antineopl astic Agents, Alkylatin g Myeloabl	SR-05000001667	B1118292
SR-01000075680-15	NCI DTP Drug Discovery	NSC296961	12	4	6	214.22296	-3.740342	-3.7830431	DB01143	Amifostine	For reduction in the cumulati ve renal toxicity	A phosphor othioate proposed as a radiation	The thiol metabolit e is responsi ble for most of	Amifostin e is an organic thiophos phate cytoprote	KYW	20537-88 6 104087 08-3 59178-37 9 63717-27-1	Amifostine	Antineopl astic For reduction in the cumulati ve renal	Radiation-Protectiv e Agents The thiol metabolit	SR-01000075680	B1118292
SR-01000941587-4	NCI DTP Drug Discovery	NSC9706	15	0	6	204.23177	1.701647	1.36453254							NEH	51-18-3	Triethylenemelamine	Antineopl astic	Antineopl astic Agents, Alkylatin g	SR-01000941587	B1118292
SR-01000076253-6	NCI DTP Drug Discovery	NSC241240	13	2	6	371.24848	1.0637	1.0637	DB00958	Carboplatin	For the initial treatment of advanced ovarian	An organopl atinum compoun d that possesse	Alkylatin g agents work by three different mechanis	Carbopla tin is an antineopl astic in the class of						SR-01000076253	B1118292
SR-05000001460-11	NCI DTP Drug Discovery	NSC718781	29	1	7	393.43572	3.200988	3.2003289	DB00530	Erlotinib	For the treatment of patients with locally	Erlotinib hydrochl oride (trade name Tarceva,	The mechanis m of clinical antitumo r action		LBX	183319-69-9 183321-74-6	erlotinib	Antineopl astic For the treatment of patients	Protein Kinase Inhibitor s The mechanis m of	SR-05000001460	B1118292
SR-02000000903-7	NCI DTP Drug Discovery	NSC138783	23	1	4	358.26283	1.664995	0.95016145							LFJ	3543-75-7 16506-27-7	Bendamustine	Antineopl astic	Antineopl astic Agents	SR-02000000903	B1118292
SR-05000174495-2	NCI DTP Drug Discovery	NSC756644	24	3	2	323.36412	2.45449	0.28046801													B1118292
SR-01000941568-4	NCI DTP Drug Discovery	NSC761431	33	2	4	489.92212	4.622483	4.26943119	DB08881	Vemurafenib	Treatmen t of unresect able or metastati c	Vemurafe nib is a BRAF enzyme inhibitor develope	Vemurafe nib is an orally available inhibitor of							SR-01000941568	B1118292
SR-05000022967-3	NCI DTP Drug Discovery	NSC758487	39	1	5	532.55948	4.965714	4.24530392													B1118292
SR-01000075919-16	NCI DTP Drug Discovery	NSC32065	5	3	2	76.05466	-1.367016	-1.3677942	DB01005	Hydroxyurea	For managem ent of melanom a, resistant	An antineopl astic agent that inhibits	Hydroxyu rea is converte d to a free radical	Hydroxyu rea has dose-depender t synergist						SR-01000075919	B1118292
SR-01000799142-15	NCI DTP Drug Discovery	NSC755	10	2	4	152.17705	0.199946	-0.8795669	DB01033	Mercaptopurine	For remissio n induction and maintena	An antimeta bolite antineopl astic agent	Mercapto purine competes with hypoxant hine and	Mercapto purine is one of a large series of purine	MAR	6112-76-1 50-44-2 7487-93-6 39454-94-9 5759-99-	Mercaptopurine	Antineopl astic For remissio n induction and	Antimeta bolites Antimeta bolites, Antineopl astic	SR-01000799142	B1118292
SR-01000076102-23	NCI DTP Drug Discovery	NSC747974	34	2	5	473.58327	5.69263	5.33663291	DB00481	Raloxifene	For the preventic n and treatment of osteopor	A second generatic n selective estrogen receptor	Raloxifen e binds to estrogen receptors ,	Raloxifen e, a selective estrogen receptor modulatc	PBC	82640-04 8 84449-90-1	raloxifene	Bone Resorptic n Inhibitor For the preventic	Bone Density Conserva tion Agents Estrogen	SR-01000076102	B1118292
SR-01000075350-17	NCI DTP Drug Discovery	NSC125973	62	4	14	853.90614	3.53884	3.53836876												SR-01000075350	B1118292
SR-01000780565-7	NCI DTP Drug Discovery	NSC266046	15	4	6	399.30163	-0.007974	-2.7383267	DB00526	Oxaliplatin	Used in combinat ion with infusiona l 5-FU/LV, is	Oxaliplat in is a platinum based chemothe rapy drug	Oxaliplat in undergoe s nonenzy matic	Oxaliplat in selectivel y inhibits the synthesis						SR-01000780565	B1118292
SR-05000001598-12	NCI DTP Drug Discovery	NSC45388	13	2	5	182.1832	-0.425889	-1.3159576	DB00851	Dacarbazine	For the treatment of metastati c malignan	An antineopl astic agent. It has significa	The mechanis m of action is not known,	Dacarbaz ine is a synthetic analog of naturally occurring	KWL	4342-03-4 37626-23-6 17925-90 5 64038-56-8	dacarbazine	Antineopl astic For the treatment of metastati	Antineopl astic Agents, Alkylatin g The mechanis	SR-05000001598	B1118292
SR-05000002138-12	NCI DTP Drug Discovery	NSC18509	9	2	4	131.12985	-3.252645	-3.3702925	DB00855	Aminolevulinic acid	Aminolev ulinic acid plus blue light illuminat ion using	A compoun d produced from succinyl-	Accordin g to the presume d mechanis m of	The metaboli sm of aminolev ulinic acid	LIL	5451-09-2 106-60-5	Pentanoic acid, 5-amino-4-oxo-	Antineopl astic Aminolev ulinic acid plus blue light	Photosen sitizing Agents Accordin g to the presume	SR-05000002138	B1118292
SR-01000941590-5	NCI DTP Drug Discovery	NSC764134	35	2	6	519.5624	5.459618	5.0990273	DB08912	Dabrafenib	Dabrafen ib is indicated for the treatment of	Dabrafen ib mesylate is a reversibl e ATP-	Dabrafen ib is an inhibitor of some mutated forms of	Dabrafen ib caused an inhibitio n of phosphor						SR-01000941590	B1118292
SR-05000174532-2	NCI DTP Drug Discovery	NSC761385	35	1	5	485.49814	6.756368	6.75166475													B1118292
SR-05000705172-1	NCI DTP Drug Discovery	NSC788948	26	1	9	370.31141	-1.091866	-1.112795													B1118292
SR-05000705153-1	NCI DTP Drug Discovery	NSC764040	36	1	5	482.61655	4.887412	4.75015849													B1118292
SR-05000705111-1	NCI DTP Drug Discovery	NSC754355	24	2	3	320.38829	2.46805	-0.1204052													B1118292
SR-01000947329-3	NCI DTP Drug Discovery	NSC776422	38	3	8	558.13513	5.793174	3.37520804	DB09063	Ceritinib	Ceritinib is a kinase inhibitor indicated for the	Ceritinib is used for the treatment of adults with	Ceritinib inhibits Anaplasti c lymphom a kinase							SR-01000947329	B1118292
SR-01000941574-4	NCI DTP Drug Discovery	NSC755986	27	1	4	421.29706	3.928932	3.92876729	DB08828	Vismodegib	Vismodeg ib is used for treating locally advanced	Vismodeg ib inhibits the hedgehog signallin	Mutation s of the Hedgehog pathway may results in	Vismodeg ib selectivel y binds to and inhibits						SR-01000941574	B1118292
SR-05000001858-6	NCI DTP Drug Discovery	NSC25154	16	0	2	356.05423	0.410781	0.41078134	DB00236	Pipobroman	For the treatment of polycyth aemia vera and	An antineopl astic agent that acts by	The mechanis m of action is uncertain but	Pipobro man is an antineopl astic agent.	UEE	54-91-1	Abbott brand of pipobroman	Antineopl astic For the treatment of polycyth	Antineopl astic Agents, Alkylatin g The mechanis	SR-05000001858	B1118292
SR-00000000529-9	NCI DTP Drug Discovery	NSC747971	32	3	4	464.82494	4.34333	4.34329875	DB00398	Sorafenib	Sorafenib is indicated for the treatment of	Sorafenib (rINN), marketed as Nexavar by Bayer,	Sorafenib interacts with multiple intracell ular	No large changes in QTc interval were observed	OSG	475207-59-1 284461-73-0	Sorafenib tosilate	Antineopl astic For the treatment of patients	Antineopl astic Agents Sorafenib interacts with	SR-00000000529	B1118292
SR-01000763672-13	NCI DTP Drug Discovery	NSC609699	31	2	7	421.44581	-0.357095	-0.9559677	DB01030	Topotecan	For the treatment of advanced ovarian cancer in	An antineopl astic agent used to treat	Topoteca n has the same mechanis m of action as	Topoteca n, a semi-synthetic derivativ e of camptoth	TQE	119413-54-6 123948-87-8	Topotecan	Antineopl astic For the treatment of advanced	Antineopl astic Agents Enzyme Inhibitor s	SR-01000763672	B1118292
SR-01000075737-17	NCI DTP Drug Discovery	NSC26271	14	1	2	261.08596	0.096548	0.09654607	DB00531	Cyclophosphamide	Cyclopho sphamid e is indicated for the treatment	Precurso r of an alkylatin g nitrogen mustard	Alkylatin g agents work by three different mechanis	Cyclopho sphamid e is an antineopl astic in the class	NGX	50-18-0 6055-19-2 60007-95-6 60007-96 7 60030-	cyclophosphamide	Antineopl astic For managem ent of malignan t	Antineopl astic Agents, Alkylatin g Antirheu	SR-01000075737	B1118292
SR-01000003023-12	NCI DTP Drug Discovery	NSC628503	58	5	14	807.87922	2.916791	2.91667076	DB01248	Docetaxel	For the treatment of patients with locally	Docetaxe l is a clinically well establish ed anti-	Docetaxe l interferes with the normal function	Docetaxe l is a taxoid antineopl astic agent. It	LRP	114977-28-5 148408-66-6	docetaxel	Antineopl astic For the treatment of patients	Antimala rials Antineopl astic Agents, Phytogen	SR-01000003023	B1118292
SR-01000941593-3	NCI DTP Drug Discovery	NSC761388	36	6	8	502.78195	-0.433592	-9.6137545	DB06809	Plerixafor	Used in combinat ion with granuloc yte-colony	Plerixafo r is a hematop oietic stem cell mobilizer	Plerixafo r inhibits the CXCR4 chemokin e	Plerixafo r is a bicyclam derivativ e that antagoni						SR-01000941593	B1118292
SR-01000000258-14	NCI DTP Drug Discovery	NSC71423	28	0	4	384.50847	3.723744	3.7237443	DB00351	Megestrol acetate	For the treatment of anorexia, cachexia, or an	17-Hydroxy-6-methylpr egna-3,6-diene-	The precise mechanis m by which megestro	Megestro l is a synthetic progestin and has the same	TUR	595-33-5 51154-23 5 79030-51-6 8064-66-2	Megestrol acetate	Progestogen	antineoplastic	SR-01000000258	B1118292
SR-01000076001-24	NCI DTP Drug Discovery	NSC279836	32	8	10	444.48091	0.754485	-1.0929221	DB01204	Mitoxantrone	For the treatment of secondar y (chronic)	An anthrace nedione-derived antineopl astic	Mitoxant rone, a DNA-reactive agent that	Mitoxant rone has been shown in vitro to inhibit B	LCA	70476-82 3 65271-80-9 70711-41 0 137635 96-2	mitoxantrone	Antineopl astic For the treatment of secondar	Analgesic s Antineopl astic Agents Mitoxant	SR-01000076001	B1118292
SR-01000629727-15	NCI DTP Drug Discovery	NSC45923	16	0	3	216.18952	1.784812	1.78481175	DB00553	Methoxsalen	For the treatment of psoriasis and vitiligo	A naturally occurring furocoum arin compoun	After activatio n it binds preferent ially to the	Methoxs alen selectivel y inhibits the synthesis	PDL	298-81-7 12692-94 3	7H-furo(3,2-g)(1)benz opyran-7 one, 9-methoxy-	Dermatol ogic For the treatment of psoriasis	Antipsori atic Agents After activatio n it binds	SR-01000629727	B1118292

SR-01000941591-2	>	5.E-6	32.49	4.84	0.11	-2.1E+0	-2.1E+0	>	5.E-6	12.54	4.47	0.11	-2.1E+0	-2.1E+0	>	5.E-6	5.47	5.82		0.11	-2.1E+0	-2.1E+0
SR-05000000373-14	>	5.E-6	20.74	3.23	0.39	3.30	3.31	>	5.E-6	35.08	2.79	0.39	3.30	3.31	>	5.E-6	48.70	0.86		0.39	3.30	3.31
SR-01000947328-3	>	5.E-6	22.48	0.98	0.22	1.67	1.73	>	5.E-6	3.94	1.78	0.22	1.67	1.73	>	5.E-6	8.69	6.09		0.22	1.67	1.73
SR-05000001855-9	>	5.E-6	3.39	8.37	0.67	6.33	6.33	>	5.E-6	3.33	6.39	0.67	6.33	6.33	>	5.E-6	30.30	59.00		0.67	6.33	6.33
SR-01000941575-5	>	5.E-6	6.02	6.40	0.25	1.73	4.35	>	5.E-6	17.69	3.64	0.25	1.73	4.35	>	5.E-6	23.71	59.24		0.25	1.73	4.35
SR-01000930807-4	>	5.E-6	8.60	3.39	0.49	3.15	3.15	>	5.E-6	5.49	3.61	0.49	3.15	3.15	>	5.E-6	1.88	5.57		0.49	3.15	3.15
SR-00000000462-6	>	5.E-6	9.51	15.27	0.25	0.76	2.49	>	5.E-6	4.30	2.83	0.25	0.76	2.49	>	5.E-6	6.68	4.20		0.25	0.76	2.49
SR-01000941571-6	>	5.E-6	24.21	5.88	0.22	0.82	0.82	>	5.E-6	2.70	8.88	0.22	0.82	0.82	>	5.E-6	19.31	4.76		0.22	0.82	0.82
SR-05000001983-6	>	5.E-6	6.34	16.52	0.74	6.18	6.20	>	5.E-6	5.73	5.56	0.74	6.18	6.20	>	5.E-6	7.99	4.87		0.74	6.18	6.20
SR-05000174675-2	>	5.E-6	33.64	7.54	0.17	2.53	4.59	>	5.E-6	47.85	5.01	0.17	2.53	4.59	=	1.1E-6	74.76	1.99	3.30	0.19	3.19	5.25
SR-01000947686-3	>	5.E-6	5.18	6.34	0.22	2.53	4.01	>	5.E-6	2.83	0.87	0.22	2.53	4.01	>	5.E-6	-883.8E-3	8.51		0.22	2.53	4.01
SR-01000000239-30	>	5.E-6	6.89	7.36	0.34	0.29	2.66	>	5.E-6	3.74	4.57	0.34	0.29	2.66	>	5.E-6	-469.9E-3	4.11		0.34	0.29	2.66
SR-05000001945-9	>	5.E-6	13.40	20.46	0.31	7.25	10.43	>	5.E-6	6.71	6.77	0.31	7.25	10.43	>	5.E-6	1.97	1.16		0.31	7.25	10.43
SR-05000002022-9	>	5.E-6	6.17	3.44	0.53	5.21	5.21	>	5.E-6	3.44	0.58	0.53	5.21	5.21	>	5.E-6	-4.E+0	1.19		0.53	5.21	5.21
SR-01000075751-15	>	5.E-6	8.35	4.65	0.41	-808.5E-3	-808.5E-3	>	5.E-6	3.31	3.33	0.41	-808.5E-3	-808.5E-3	>	5.E-6	-1.6E+0	6.43		0.41	-808.5E-3	-808.5E-3
SR-01000075662-21	>	5.E-6	25.83	20.04	0.44	8.37	8.37	>	5.E-6	14.38	7.32	0.44	8.37	8.37	>	5.E-6	-3.5E+0	3.33		0.44	8.37	8.37
SR-01000939639-7	>	5.E-6	8.62	18.53	0.24	1.75	1.75	>	5.E-6	3.00	2.40	0.24	1.75	1.75	>	5.E-6	-1.4E+0	2.22		0.24	1.75	1.75
SR-01000837528-12	>	5.E-6	11.86	10.78	0.29	1.29	1.29	>	5.E-6	6.68	3.38	0.29	1.29	1.29	>	5.E-6	0.34	19.70		0.29	1.29	1.29
SR-05000174566-2	>	5.E-6	16.45	0.78	0.12	-1.5E+0	-1.6E+0	>	5.E-6	4.97	0.67	0.12	-1.5E+0	-1.6E+0	>	5.E-6	-3.1E+0	2.27		0.12	-1.5E+0	-1.6E+0
SR-01000075736-14	>	5.E-6	21.11	20.53	0.62	4.28	4.28	>	5.E-6	3.67	3.89	0.62	4.28	4.28	>	5.E-6	-3.1E+0	1.11		0.62	4.28	4.28
SR-01000763561-14	>	5.E-6	4.85	8.41	0.20	0.92	1.85	>	5.E-6	7.21	1.05	0.20	0.92	1.85	>	5.E-6	-3.2E+0	5.02		0.20	0.92	1.85
SR-05000001667-5	>	5.E-6	3.89	10.04	0.39	5.05	5.05	>	5.E-6	0.83	1.93	0.39	5.05	5.05	>	5.E-6	16.56	3.92		0.39	5.05	5.05
SR-01000075680-15	>	5.E-6	9.25	6.07	0.62	9.04	9.08	>	5.E-6	4.47	2.74	0.62	9.04	9.08	>	5.E-6	-4.9E+0	0.68		0.62	9.04	9.08
SR-01000941587-4	>	5.E-6	24.92	29.59	0.49	3.60	3.94	>	5.E-6	5.09	4.03	0.49	3.60	3.94	>	5.E-6	8.63	4.41		0.49	3.60	3.94
SR-01000076253-6	>	5.E-6	7.11	5.11	0.57	4.24	4.24	>	5.E-6	2.24	3.65	0.57	4.24	4.24	>	5.E-6	-315.E-3	1.81		0.57	4.24	4.24
SR-05000001460-11	>	5.E-6	34.81	26.85	0.26	2.10	2.10	>	5.E-6	16.21	0.11	0.26	2.10	2.10	>	5.E-6	11.58	10.78		0.26	2.10	2.10
SR-02000000903-7	>	5.E-6	7.46	3.86	0.32	3.64	4.35	>	5.E-6	2.99	3.92	0.32	3.64	4.35	>	5.E-6	-5.4E+0	4.86		0.32	3.64	4.35
SR-05000174495-2	>	5.E-6	10.38	19.57	0.31	2.85	5.02	>	5.E-6	8.17	4.36	0.31	2.85	5.02	>	5.E-6	-353.2E-3	1.33		0.31	2.85	5.02
SR-01000941568-4	>	5.E-6	1.14	1.93	0.22	0.68	1.03	>	5.E-6	5.07	1.85	0.22	0.68	1.03	>	5.E-6	-6.5E+0	3.82		0.22	0.68	1.03
SR-05000022967-3	>	5.E-6	0.47	14.88	0.19	0.34	1.06	>	5.E-6	1.11	5.92	0.19	0.34	1.06	>	5.E-6	3.97	0.59		0.19	0.34	1.06
SR-01000075919-16	>	5.E-6	21.93	19.06	1.48	6.67	6.67	>	5.E-6	5.23	3.98	1.48	6.67	6.67	>	5.E-6	-2.7E+0	2.16		1.48	6.67	6.67
SR-01000799142-15	>	5.E-6	13.50	17.97	0.74	5.10	6.18	>	5.E-6	19.18	6.22	0.74	5.10	6.18	>	5.E-6	45.65	2.29		0.74	5.10	6.18
SR-01000076102-23	>	5.E-6	13.93	17.55	0.22	-390.7E-3	-34.7E-3	>	5.E-6	5.10	2.02	0.22	-390.7E-3	-34.7E-3	>	5.E-6	-5.9E+0	4.68		0.22	-390.7E-3	-34.7E-3
SR-01000075350-17	>	5.E-6	41.08	2.84	0.12	1.76	1.76	>	5.E-6	41.60	13.79	0.12	1.76	1.76	=	21.3E-9	61.33	1.36	1.73	0.17	4.13	4.13
SR-01000780565-7	>	5.E-6	8.36	4.45	0.49	5.31	8.04	>	5.E-6	4.03	2.33	0.49	5.31	8.04	>	5.E-6	-1.5E+0	2.78		0.49	5.31	8.04
SR-05000001598-12	>	5.E-6	12.89	20.28	0.57	5.73	6.62	>	5.E-6	5.36	4.80	0.57	5.73	6.62	>	5.E-6	1.37	4.36		0.57	5.73	6.62
SR-05000002138-12	>	5.E-6	9.67	3.19	0.82	8.55	8.67	>	5.E-6	31.25	59.00	0.82	8.55	8.67	>	5.E-6	-5.9E+0	4.54		0.82	8.55	8.67
SR-01000941590-5	>	5.E-6	1.99	3.22	0.21	-157.7E-3	0.20	>	5.E-6	6.72	5.85	0.21	-157.7E-3	0.20	>	5.E-6	-7.E+0	7.71		0.21	-157.7E-3	0.20
SR-05000174532-2	>	5.E-6	16.72	24.79	0.21	-1.5E+0	-1.4E+0	>	5.E-6	4.70	1.47	0.21	-1.5E+0	-1.4E+0	>	5.E-6	2.86	2.27		0.21	-1.5E+0	-1.4E+0
SR-05000705172-1	>	5.E-6	16.56	12.71	0.29	6.39	6.41	>	5.E-6	5.16	2.40	0.29	6.39	6.41	>	5.E-6	7.13	4.04		0.29	6.39	6.41
SR-05000705153-1	>	5.E-6	17.41	26.44	0.21	0.41	0.55	>	5.E-6	6.54	3.14	0.21	0.41	0.55	>	5.E-6	-3.1E+0	2.57		0.21	0.41	0.55
SR-05000705111-1	>	5.E-6	9.74	8.01	0.31	2.83	5.42	>	5.E-6	4.55	2.23	0.31	2.83	5.42	>	5.E-6	8.99	11.70		0.31	2.83	5.42
SR-01000947329-3	>	5.E-6	3.32	6.12	0.20	-491.3E-3	1.93	>	5.E-6	10.44	3.36	0.20	-491.3E-3	1.93	>	5.E-6	14.41	0.71		0.20	-491.3E-3	1.93
SR-01000941574-4	>	5.E-6	4.20	24.50	0.27	1.37	1.37	>	5.E-6	5.14	4.63	0.27	1.37	1.37	>	5.E-6	11.91	3.24		0.27	1.37	1.37
SR-05000001858-6	>	5.E-6	-666.E-3	7.65	0.46	4.89	4.89	>	5.E-6	4.00	2.24	0.46	4.89	4.89	>	5.E-6	7.45	21.29		0.46	4.89	4.89
SR-00000000529-9	>	5.E-6	16.86	21.12	0.23	0.96	0.96	>	5.E-6	8.55	4.54	0.23	0.96	0.96	>	5.E-6	13.55	9.29		0.23	0.96	0.96
SR-01000763672-13	>	5.E-6	2.42	13.88	0.24	5.66	6.26	>	5.E-6	6.70	2.67	0.24	5.66	6.26	>	5.E-6	-2.4E+0	3.82		0.24	5.66	6.26
SR-01000075737-17	>	5.E-6	9.45	21.53	0.53	5.21	5.21	>	5.E-6	5.26	3.11	0.53	5.21	5.21	>	5.E-6	-5.3E+0	3.33		0.53	5.21	5.21
SR-01000003023-12	>	5.E-6	46.57	19.46	0.13	2.39	2.39	>	5.E-6	41.40	5.78	0.13	2.39	2.39	>	6.8E-9	56.11	0.96		0.20	5.25	5.25
SR-01000941593-3	>	5.E-6	13.03	20.79	0.21	5.74	14.92	>	5.E-6	8.16	4.77	0.21	5.74	14.92	>	5.E-6	3.23	4.58		0.21	5.74	14.92
SR-01000000258-14	>	5.E-6	11.04	21.02	0.27	1.58	1.58	>	5.E-6	5.21	4.22	0.27	1.58	1.58	>	5.E-6	-2.2E+0	4.58		0.27	1.58	1.58
SR-01000076001-24	>	5.E-6	30.93	21.31	0.23	4.55	6.39	>	5.E-6	45.06	4.12	0.23	4.55	6.39	=	569.3E-9	69.98	0.88	13.03	0.27	5.49	7.34
SR-01000629727-15	>	5.E-6	4.79	8.90	0.46	3.52	3.52	>	5.E-6	5.06	3.42	0.46	3.52	3.52	>	5.E-6	7.03	29.03		0.46	3.52	3.52

SR-01000941578-4	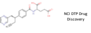	NCI DTP Drug Discovery	NSC754230	35	5	11	477.47262	0.615166	-5.7050032	DB06813	Pralatrexate	Treatment of relapsed or refractory	Pralatrexate is an antimetabolite for the treatment	The selectivity of pralatrexate for cancer	Pralatrexate is a 10-deazaaminopterin analogue						SR-01000941578	B1118292
SR-01000075881-13	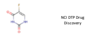	NCI DTP Drug Discovery	NSC19893	9	2	2	130.07722	-0.655848	-0.810272	DB00544	Fluorouracil	For the topical treatment of multiple actinic or	A pyrimidine analog that is an antineopl	The precise mechanism of action has not	Fluorouracil is an antineoplastic antimetabolite. Anti-	UUX	51-21-8 10318-20 4 57050-04-1 57172-36 8 68021-	Fluorouracil	Antineoplastic For the topical treatment of	Antimetabolites Antimetabolites, Antineoplastic	SR-01000075881	B1118292
SR-01000000117-18	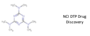	NCI DTP Drug Discovery	NSC13875	15	0	6	210.27941	2.216222	1.71631938	DB00488	Altretamine	For use as a single agent in the palliative	An alkylating agent proposed as an antineopl	The precise mechanism by which altretami	Altretamine is a novel antineoplastic agent.	RKT	645-05-6 15468-34 5 2975-00-0	Altretaminum	Antineoplastic For use as a single agent in the	Antineoplastic Agents, Alkylating The precise	SR-01000000117	B1118292
SR-01000597029-4	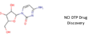	NCI DTP Drug Discovery	NSC63878	17	4	7	243.21662	-2.797517	-2.7975203													B1118292
SR-01000597562-4	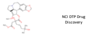	NCI DTP Drug Discovery	NSC758253	39	2	10	545.62125	1.884671	-0.1241306	DB04865	Homoharringtonine	Used in patients who are intolerant and/or resistant	Homoharringtonine, AKA HHT or omacetaxine	Homoharringtonine inhibits protein synthesis by not	The pharmacodynamics of homoharringtonin	SMW	26833-87 4 88179-67-3	4'-Methyl 2'r-hydroxy-2'-(4-hydroxy-4-	Antineoplastic	Antineoplastic Agents, Phytogenic Angiogen	SR-01000597562	B1118292
SR-05000001664-7	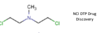	NCI DTP Drug Discovery	NSC762	8	0	1	156.05354	1.519149	1.49906237	DB00888	Mechlorethamine	For the palliative treatment of Hodgkin's disease	A vesicant and necrotizing irritant	Alkylating agents work by three different mechanis	Mechlorethamine also known as mustine, nitrogen	TPK	51-75-2 126-85-2 302-70-5 55-86-7 159923-90-7	Chlormethine	Antineoplastic For the palliative treatment of	Alkylating Agents Antineoplastic Agents, Alkylatin	SR-05000001664	B1118292
SR-05000174588-2	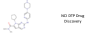	NCI DTP Drug Discovery	NSC778909	32	2	7	434.53729	2.379943	0.89095841													B1118292
SR-05000001472-9	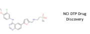	NCI DTP Drug Discovery	NSC745750	40	2	7	581.05754	4.638509	4.42501139	DB01259	Lapatinib	Indicated in combination with capecitabine for	Lapatinib is an anti cancer drug developed by	Lapatinib is a 4-anilinoquinazoline kinase inhibitor	Lapatinib is a small molecule and a member	NSK	388082-78-8 231277-92-2	lapatinib	Antineoplastic Indicated in combination with	Tyrosine kinase inhibitors Antineoplastic	SR-05000001472	B1118292
SR-01000076184-22	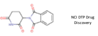	NCI DTP Drug Discovery	NSC66847	19	1	4	258.22949	0.015707	0.01567983	DB01041	Thalidomide	For the acute treatment of the cutaneous	A piperidinyl isoindole originally	In patients with erythema nodosum leprosum	Thalidomide is an immunomodulatory agent with a	MDT	50-35-1 731-40-8 841-67-8 14088-68 7 2614-06-4	N-(2,6-dioxo-3-piperidyl)phthalimide	Immunomodulator For the acute treatment of the	Angiogenesis Inhibitors Immunosuppressi	SR-01000076184	B1118292
SR-01000941586-5	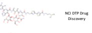	NCI DTP Drug Discovery	NSC125066	96	20	29	1415.55175	-9.710223	-8.9322067							MNH	9041-93-4 11056-06-7 1400-95-9 37208-04-1	Bleomycin	Antineoplastic For palliative treatment in the managem	Antineoplastic Agents Although the exact mechanis	SR-01000941586	B1118292
SR-01000721911-6	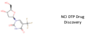	NCI DTP Drug Discovery	NSC75520	20	3	5	296.19994	-0.754626	-0.9644963	DB00432	Trifluridine	Ophthalmic solution for the treatment of primary	An antiviral derivative of thymidine used	The mechanism of action of trifluridine has	Trifluridine is a fluorinated pyrimidine	UTN	70-00-8	Trifluridine	Antiviral Ophthalmic solution for the treatment	Antimetabolites Antiviral Agents The mechanis	SR-01000721911	B1118292
SR-01000930565-7	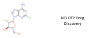	NCI DTP Drug Discovery	NSC606869	20	3	7	303.67744	-0.291322	-0.291324	DB00631	Clofarabine	For the treatment of pediatric patients 1 to 21	Clofarabine is a purine nucleoside antimeta	Clofarabine is metabolized intracellularly to	Clofarabine is a purine nucleoside antimeta	PYV	123318-82-1	clofarabine	Antineoplastic For the treatment of pediatric	Antineoplastic Agents Antimetabolites Clofarabi	SR-01000930565	B1118292
SR-01000597410-4	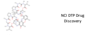	NCI DTP Drug Discovery	NSC3053	90	5	18	1255.41704	-0.096882	-0.0971764	DB00970	Dactinomycin	For the treatment of Wilms' tumor, childhood	A compound composed of a two	Good evidence exists that this drug binds strongly,	Generally, the actinomycins exert an inhibitor						SR-01000597410	B1118292
SR-01000931150-7	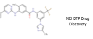	NCI DTP Drug Discovery	NSC747599	39	2	6	529.51578	5.36154	5.33148059	DB04868	Nilotinib	For the potential treatment of various leukemia	Nilotinib, also known as AMN107, is a tyrosine	Chronic myelogenous leukaemia (CML) is caused	Nilotinib is a transduction inhibitor that						SR-01000931150	B1118292
SR-01000076254-7	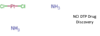	NCI DTP Drug Discovery	NSC119875	5	2	2	300.04504	1.299	1.299	DB00515	Cisplatin	For the treatment of metastatic testicular	Cisplatin cisplatinum or cis diamminedichloroplatinum	Alkylating agents work by three different mechanis	Cisplatin is an antineoplastic in the class of						SR-01000076254	B1118292
SR-01000611320-11	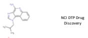	NCI DTP Drug Discovery	NSC369100	18	1	3	240.30364	2.650669	2.64643826	DB00724	Imiquimod	For the topical treatment of clinically typical,	Imiquimod is an immune response modifier that acts	Imiquimod's mechanism of action is via	Imiquimod is an immune response modifier that acts	TOU	99011-02-6	imiquimod	Antiviral For the topical treatment of clinically	Adjuvants, Immunologic Antineoplastic	SR-01000611320	B1118292
SR-01000879991-4	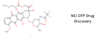	NCI DTP Drug Discovery	NSC246131	51	5	13	723.64374	4.486147	3.50592857	DB00385	Valrubicin	For the treatment of cancer of the bladder.	Valrubicin (N-trifluoroacetyladriamycin-14-	Valrubicin is an anthracycline that affects a variety of	Valrubicin is a semisynthetic analog of the	ROD	56124-62-0	Valrubicin	Antineoplastic For the treatment of cancer of the	Antineoplastic Agents Valrubicin is an anthracy	SR-01000879991	B1118292
SR-01000883935-5	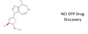	NCI DTP Drug Discovery	NSC218321	19	4	7	268.26914	-2.016391	-2.9345256	DB00552	Pentostatin	For the treatment of hairy cell leukaemia	A potent inhibitor of adenosine deamina	Pentostatin is a potent transition state inhibitor	Pentostatin is an antineoplastic antimetabolite used in	OPS	53910-25 1 63677-95-2 59979-24 7 69196-00-5	pentostatin	Antineoplastic For the treatment of hairy cell	Antibiotics, Antineoplastic Enzyme Inhibitor	SR-01000883935	B1118292
SR-05000174531-2	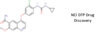	NCI DTP Drug Discovery	NSC755980	30	3	5	426.85295	2.521607	2.51743845													B1118292
SR-05000174592-2	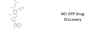	NCI DTP Drug Discovery	NSC779217	37	2	7	499.60731	4.486981	3.00856316													B1118292
SR-01000763375-11	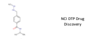	NCI DTP Drug Discovery	NSC77213	16	3	3	221.29876	0.985734	0.97892093	DB01168	Procarbazine	For use with other anticancer drugs for the	An antineoplastic agent used primarily	The precise mode of cytotoxic action of procarba	Procarbazine is an antineoplastic in the class of	OBW	366-70-1 671-16-9 13309-74 5 18969-59-0 98600-80	Procarbazine hydrochloride	Antineoplastic For use with other anticancer drugs	Antineoplastic Agents The precise mode of	SR-01000763375	B1118292
SR-01000898799-5	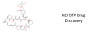	NCI DTP Drug Discovery	NSC683864	73	4	16	1030.28708	7.129959	7.12877626	DB06287	Temsirolimus	For the treatment of renal cell carcinoma (RCC).	Temsirolimus is an intravenous drug for the treatment	Temsirolimus is an inhibitor of mTOR (mammalian target		VRX	162635-04-3	temsirolimus	Antineoplastic	Antineoplastic Agents Protein Kinase Inhibitor	SR-01000898799	B1118292
SR-01000003063-17	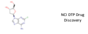	NCI DTP Drug Discovery	NSC105014	19	3	7	285.68698	-0.281775	-0.2817757	DB00242	Cladribine	For the treatment of active hairy cell leukemia (leukemic	An antineoplastic agent used in the	Cladribine is structurally related to fludarabi	Cladribine is a synthetic purine nucleoside that						SR-01000003063	B1118292

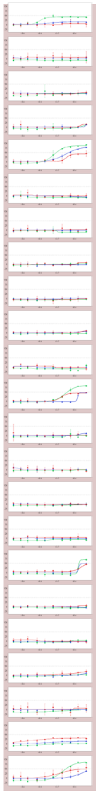

SR-01000941578-4	>	5.E-6	7.49	4.22		0.21	4.69	11.01	>	5.E-6	17.76	11.46		0.21	4.69	11.01	>	5.E-6	47.92	8.11		0.21	4.69	11.01
SR-01000075881-13	>	5.E-6	15.91	18.48		0.82	5.96	6.11	>	5.E-6	6.58	2.57		0.82	5.96	6.11	>	5.E-6	-3.2E+0	2.01		0.82	5.96	6.11
SR-01000000117-18	>	5.E-6	3.87	7.95		0.49	3.09	3.59	>	5.E-6	2.96	1.08		0.49	3.09	3.59	>	5.E-6	11.10	34.70		0.49	3.09	3.59
SR-01000597029-4	>	5.E-6	16.33	4.56		0.44	8.10	8.10	>	5.E-6	14.72	3.52		0.44	8.10	8.10	>	5.E-6	15.05	1.46		0.44	8.10	8.10
SR-01000597562-4	>	5.E-6	43.86	15.72		0.19	3.42	5.43	=	486.2E-9	78.42	1.37	1.07	0.23	4.43	6.44	=	92.1E-9	91.80	1.89	1.48	0.25	5.15	7.16
SR-05000001664-7	>	5.E-6	16.16	17.58		0.93	3.78	3.80	>	5.E-6	0.52	2.54		0.93	3.78	3.80	>	5.E-6	4.00	21.15		0.93	3.78	3.80
SR-05000174588-2	>	5.E-6	10.37	21.65		0.23	2.92	4.41	>	5.E-6	3.02	6.78		0.23	2.92	4.41	>	5.E-6	0.12	18.75		0.23	2.92	4.41
SR-05000001472-9	>	5.E-6	8.90	5.28		0.19	0.66	0.88	>	5.E-6	1.84	3.25		0.19	0.66	0.88	>	5.E-6	4.41	2.06		0.19	0.66	0.88
SR-01000076184-22	>	5.E-6	-243.8E-3	9.34		0.39	5.29	5.29	>	5.E-6	2.47	8.04		0.39	5.29	5.29	>	5.E-6	-2.E+0	7.34		0.39	5.29	5.29
SR-01000941586-5	>	5.E-6	9.81	3.11		0.08	15.01	14.23	>	5.E-6	8.86	5.08		0.08	15.01	14.23	>	5.E-6	-1.5E+0	8.57		0.08	15.01	14.23
SR-01000721911-6	>	5.E-6	14.97	2.65		0.37	6.06	6.27	>	5.E-6	7.10	2.10		0.37	6.06	6.27	>	5.E-6	8.30	6.29		0.37	6.06	6.27
SR-01000930565-7	>	5.E-6	48.03	0.87		0.37	5.59	5.59	>	5.E-6	46.63	1.53		0.37	5.59	5.59	=	397.9E-9	83.50	0.62	1.40	0.45	6.69	6.69
SR-01000597410-4	>	5.E-6	15.00	44.49		0.08	5.40	5.40	>	5.E-6	11.82	4.20		0.08	5.40	5.40	>	5.E-6	8.60	24.82		0.08	5.40	5.40
SR-01000931150-7	>	5.E-6	10.72	22.13		0.19	-59.6E-3	-29.6E-3	>	5.E-6	11.81	5.22		0.19	-59.6E-3	-29.6E-3	>	5.E-6	0.33	4.13		0.19	-59.6E-3	-29.6E-3
SR-01000076254-7	>	5.E-6	5.82	0.72		1.48	4.00	4.00	>	5.E-6	3.69	2.77		1.48	4.00	4.00	>	5.E-6	-2.1E+0	3.62		1.48	4.00	4.00
SR-01000611320-11	>	5.E-6	10.40	5.73		0.41	2.65	2.66	>	5.E-6	2.82	1.09		0.41	2.65	2.66	>	5.E-6	-3.2E+0	6.49		0.41	2.65	2.66
SR-01000879991-4	>	5.E-6	44.60	3.82		0.15	0.82	1.80	>	5.E-6	47.24	4.18		0.15	0.82	1.80	=	1.9E-6	68.93	1.46	11.71	0.16	1.24	2.22
SR-01000883935-5	>	5.E-6	6.25	7.11		0.39	7.32	8.24	>	5.E-6	2.61	1.66		0.39	7.32	8.24	>	5.E-6	0.60	5.68		0.39	7.32	8.24
SR-05000174531-2	>	5.E-6	11.60	12.77		0.25	2.78	2.78	>	5.E-6	1.25	1.96		0.25	2.78	2.78	>	5.E-6	5.34	3.25		0.25	2.78	2.78
SR-05000174592-2	>	5.E-6	21.00	7.82		0.20	0.81	2.29	>	5.E-6	12.32	2.90		0.20	0.81	2.29	>	5.E-6	4.07	2.66		0.20	0.81	2.29
SR-01000763375-11	>	5.E-6	15.23	13.07		0.46	4.32	4.32	>	5.E-6	1.55	4.14		0.46	4.32	4.32	>	5.E-6	-554.8E-3	2.15		0.46	4.32	4.32
SR-01000898799-5	>	5.E-6	33.78	3.05		0.10	-1.8E+0	-1.8E+0	>	5.E-6	15.47	3.32		0.10	-1.8E+0	-1.8E+0	>	5.E-6	5.20	3.14		0.10	-1.8E+0	-1.8E+0
SR-01000003063-17	=	97.5E-6	55.62	16.61	0.99	0.30	4.29	4.29	>	5.E-6	37.84	2.93		0.39	5.58	5.58	=	336.7E-9	84.29	2.06	1.26	0.48	6.75	6.75

Supplemental figures

Figure S2 Controls used for dose-response test of the tumor organoids against the NCI library (128 compounds)

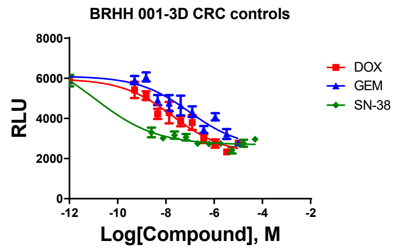

3D BRRH 001	DOX	GEM	SN-38
HillSlope	-0.41	-0.41	-0.34
IC50	2.01E-08	9.61E-08	>5.00E-06

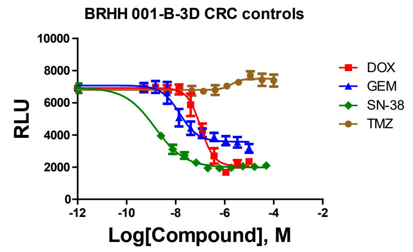

3D BRRH 001-B	DOX	GEM	SN-38	TMZ
HillSlope	-1.58	-1.16	-0.71	2.07
IC50	1.05E-07	1.47E-08	1.44E-09	1.90E-06

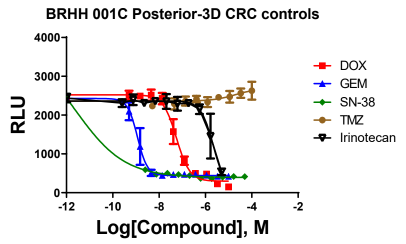

3D BRRH 001C Posterior	DOX	GEM	SN-38	TMZ	Irinotecan
HillSlope	-1.52	-2.04	-0.41	-	-1.63
IC50	5.59E-08	1.16E-09	2.53E-12	>5.00E-06	2.18E-06

BRHH 001 Run Statistics (n=3 plates)
- 128 compounds tested starting at ~5uM* in 10 point 3 fold dilution in triplicate.
- * Most common starting concentration
- Ave Z' = 0.69 ± 0.10
- Ave S:B = 98.25 ± 44.39

BRRH 001-B Run Statistics (n=3 plates)
- 128 compounds tested starting at ~5uM* in 10 point 3 fold dilution in triplicate.
- * Most common starting concentration
- Ave Z' = 0.86 ± 0.01
- Ave S:B = 135.44 ± 1.07

BRRH 001-C Run Statistics (n=3 plates)
- 128 compounds tested starting at ~5uM* in 10 point 3 fold dilution in triplicate.
- * Most common starting concentration
- Ave Z' = 0.84 ± 0.04
- Ave S:B = 155.08 ± 20.12

Figure S2: Controls used for dose-response analysis of the 3 tumor organoids against the 128-compound library

Concentration response curves of the controls run each time the individual 3D organoids were tested. Hillslope and CC50 results are shown below each graph which are color coded to match their individual curves. Analysis was done use GraphPad Prism non-linear curve fit with variable slope. Each concentration tested in each curve represents an N of 16 and error bars are shown as SD. Runs statistics are shown for each of the experiments for the individual organoids when tested vs. 128 separate NCI FDA approved oncologic drugs. The average Z's where derived from the 3 separate plates tested in each assay which included 24 control wells per plate.

Figure S3: Control for synergy combinations (Dabrafenib and Trametinib)

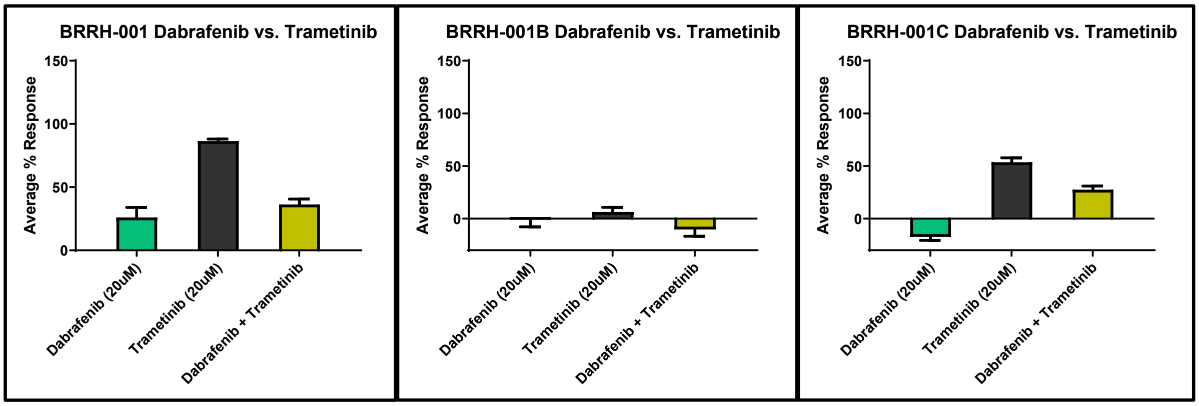

Figure S3: Controls used for synergy drug testing

In vitro drug response, derived from the 3D viability assay of patient derived GBM spheroids treated with sub IC-50 concentration (20uM) of Debrafenib, Trametinin or a combination of both shown as avg % cytotoxicity (Response). Dabrafenib and Trametinib clearly show no synergistic effects on the tumor growth. Data presented as mean±SD. Measures repeated with 6 replicate wells for individual drugs and 9 replicate wells for the combinations.

Figure S4 KEGG enrichment P-values and Volcano plots showing overview of differential expression of all the genes tested by RNAseq analysis

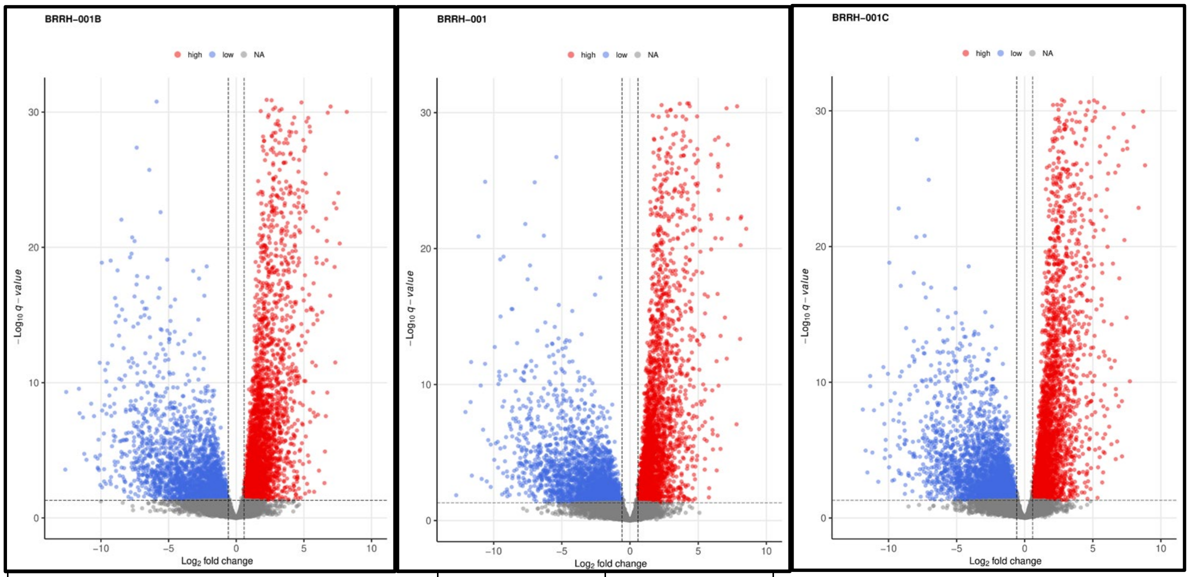

**Pathway Enrichment P-Values**	BRRH-001	BRRH-001B	BRRH-001C
EGFR tyrosine kinase	0.117	0.112	0.045
ErbB signaling pathway	0.045	0.046	0.018
Circadian rhythm	0.496	0.890	0.012
Glioma	0.045	0.206	0.021
FoxO signaling pathway	0.049	0.263	0.026
MAPK signaling pathway	0.654	0.031	0.465
Rap1 signaling pathway	0.148	0.003	0.169
Pathways in cancer	0.046	0.008	0.238
Cell cycle	5.0E-16	3.1E-12	1.1E-08
p53 signaling pathway	8.9E-06	2.5E-04	1.3E-04
DNA replication	2.3E-12	1.9E-06	4.8E-09

Figure S4: Overview of differential expression of genes in Tumor organoids BRRH-001, BRRH-001B, BRRH-001C

The threshold in the volcano plot was FDR q-value < 0.05 and |Fold Change| > 1.5. The unbalanced distribution is typical when comparing single samples against many (i.e. GTEX normal brain samples) in edgeR. KEGG Pathway Enrichment p-values between GBM organoids and GTEx healthy brains. Pathways involved in DNA replication, cell cycle, and p53 signaling are dysregulated in all three samples while the MAPK and Rap1 signaling pathways are enriched in only the first recurrence but the EGFR, ErbB, and FoxO signaling pathways are more dysregulated in the second recurrence.

Figure S5. Body weight as a measure of toxicity during tumor growth and treatment.

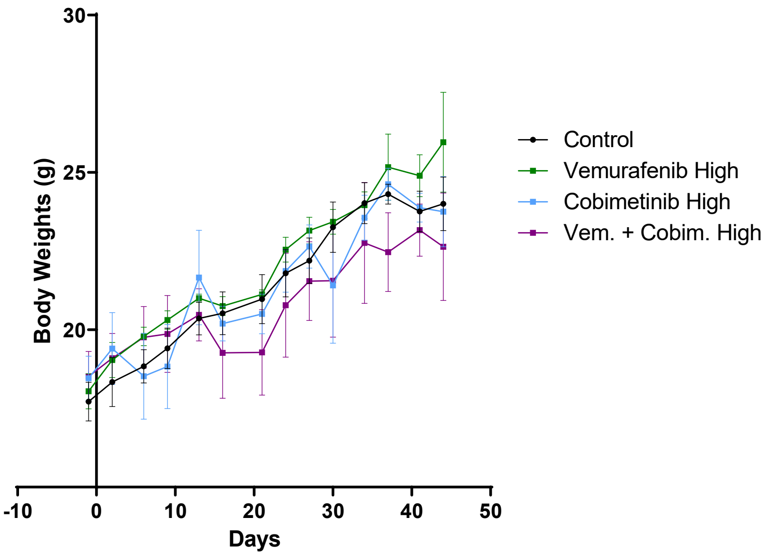

Figure S5: Body weight in mice with tumor growth and treatment.

Mice subcutaneously implanted with a patient derived spheroids cultured from a primary tumor (BRRH-001), were treated with control placebo or Cobimetinib or Vemurafenib or a combination of both). Body weights were monitored at regular intervals starting 48 hours prior to treatment. (N=4 mice in each group, all data are mean ± SEM; 2-way ANOVA and multiple comparison against control group with Bonferroni correction: Not significantly different)

Figure S6 Normality testing of tumor growth data

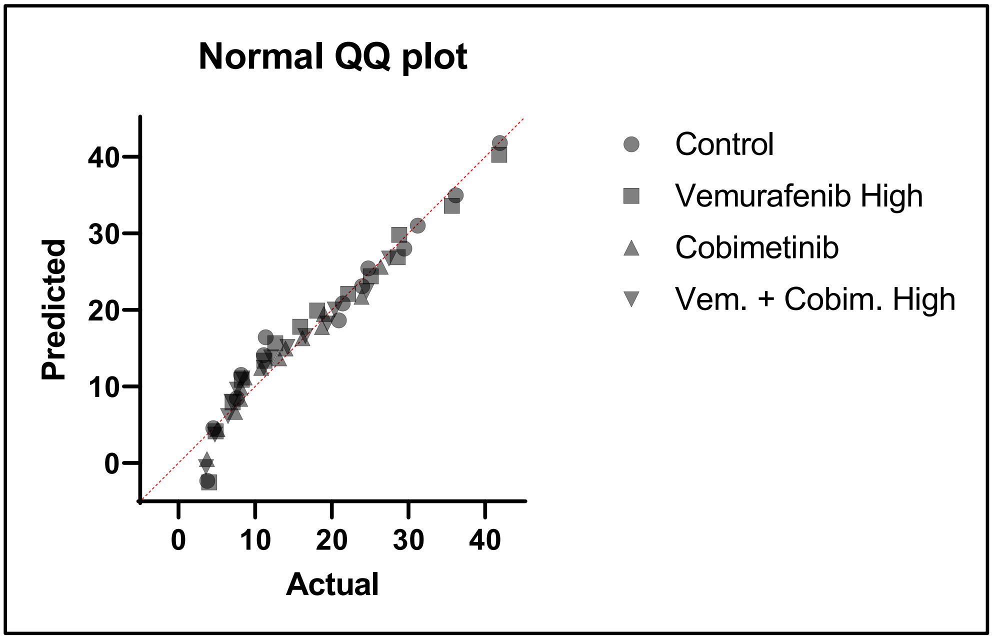

**Test for normal distribution**<br>**D'Agostino & Pearson test**				
K2	1.360	1.131	1.207	1.481
P value	0.5067	0.5681	0.5468	0.4768
Passed normality test (alpha=0.05)?	Yes	Yes	Yes	Yes
P value summary	ns	ns	ns	ns
Number of values	14	14	14	14
